# Quantitative proteomics of the tobacco pollen tube secretome identifies novel pollen tube guidance proteins important for fertilization

**DOI:** 10.1186/s13059-016-0928-x

**Published:** 2016-05-03

**Authors:** Said Hafidh, David Potěšil, Jan Fíla, Věra Čapková, Zbyněk Zdráhal, David Honys

**Affiliations:** Laboratory of Pollen Biology, Institute of Experimental Botany ASCR, Rozvojová 263, 165 02 Prague 6, Czech Republic; Research group Proteomics, CEITEC-MU, Masaryk University, Kamenice 5, 625 00 Brno, Czech Republic; Laboratory of Functional Genomics and Proteomics, National Centre for Biomolecular Research, Faculty of Science, Masaryk University, Kamenice 5, 625 00 Brno, Czech Republic

**Keywords:** Protein secretion, Pollen tube guidance, Cell-cell signaling, Double fertilization

## Abstract

**Background:**

As in animals, cell–cell communication plays a pivotal role in male–female recognition during plant sexual reproduction. Prelaid peptides secreted from the female reproductive tissues guide pollen tubes towards ovules for fertilization. However, the elaborate mechanisms for this dialogue have remained elusive, particularly from the male perspective.

**Results:**

We performed genome-wide quantitative liquid chromatography–tandem mass spectrometry analysis of a pistil-stimulated pollen tube secretome and identified 801 pollen tube-secreted proteins. Interestingly, *in silico* analysis reveals that the pollen tube secretome is dominated by proteins that are secreted unconventionally, representing 57 % of the total secretome. In support, we show that an unconventionally secreted protein, translationally controlled tumor protein, is secreted to the apoplast. Remarkably, we discovered that this protein could be secreted by infiltrating through the initial phases of the conventional secretory pathway and could reach the apoplast via exosomes, as demonstrated by co-localization with Oleisin1 exosome marker. We demonstrate that translationally controlled tumor protein-knockdown *Arabidopsis thaliana* plants produce pollen tubes that navigate poorly to the target ovule and that the mutant allele is poorly transmitted through the male. Further, we show that regulators of the endoplasmic reticulum–trans-Golgi network protein secretory pathway control secretion of *Nicotiana tabacum* Pollen tube-secreted cysteine-rich protein 2 and Lorelei-like GPI-anchor protein 3 and that a regulator of endoplasmic reticulum–trans-Golgi protein translocation is essential for pollen tube growth, pollen tube guidance and ovule-targeting competence.

**Conclusions:**

This work, the first study on the pollen tube secretome, identifies novel genome-wide pollen tube-secreted proteins with potential functions in pollen tube guidance towards ovules for sexual reproduction. Functional analysis highlights a potential mechanism for unconventional secretion of pollen tube proteins and reveals likely regulators of conventional pollen tube protein secretion. The association of pollen tube-secreted proteins with marker proteins shown to be secreted via exosomes in other species suggests exosome secretion is a possible mechanism for cell–cell communication between the pollen tube and female reproductive cells.

**Electronic supplementary material:**

The online version of this article (doi:10.1186/s13059-016-0928-x) contains supplementary material, which is available to authorized users.

## Background

The cell apoplast and the extracellular matrix provide a hub for cell–cell communication in plants. These interspaces relay secreted peptide-mediated signals to neighboring cells. In flowering plant reproduction, the pollen tubes carrying two non-motile sperm cells (male gametes) grow through the extracellular matrix of the transmitting tract tissues (TT) with the aid of female guidance signals to reach and fertilize deeply embedded female gametes [1, 2]. This molecular dialog between the pollen tube and pollen tube attractants has emerged as an important bottleneck for unfavorable fertilization and a pre-zygotic barrier for interspecies hybridization; however, its mechanism has so far remained unknown. Studies using single-cell laser ablation and genetic techniques have identified various female secreted peptides involved in pollen–pistil interactions with conserved roles across plant species [3–5]. Predominantly, they are arabinogalactan proteins, cysteine-rich polypeptides, defensin-like (DEFL) proteins, S-RNases, hydroxyproline-rich proteins, transmitting tissue-specific (TTS) proteins, class III pistil extensin-like proteins (PELPIII) and lipid transfer proteins (LTPs) [6–8]. Recently, CENTRAL CELL GUIDANCE (CCG) protein together with its interacting partners, CCG BINDING PROTEIN1 (CBP1), mediator complex (MED), and central cell-specific AGAMOUS-transcription factors, was shown to co-regulate a subset of cysteine-rich proteins (CRPs), including pollen tube attractant LURE1, that mediate pollen tube attraction [9].

A handful of proteins are known to be secreted by the male gametophyte. They include LAT52, a pollen tube-secreted ligand from tomato [10, 11]; lipid transfer protein 5 (LTP5), a homolog of Lily SCA protein [9]; thionin [12]; and HAP2 as a sperm-specific factor required for gamete fusion and blocking polytubey [13].

The discovery of pollen tube-secreted proteins that perceive female-secreted signals has been hampered by the inaccessibility of the pollen tubes within pistils and the likelihood of contamination from surrounding female tissues. We have therefore improvised a semi-in vivo technique (SIV) for tobacco pollen tube growth through the pistil to allow capture and detection of proteins secreted by the pollen tube following its penetration through the stigma and style [14]. In contrast to in vitro grown pollen tubes, SIV pollen tubes have been shown to have unique transcriptomes [15] as well as the ability to respond to synthetic pollen tube attractant peptides (LUREs) secreted by female synergid cells of *Torenia fournieri* [3, 16]. These findings emphasize the necessity for research into pollen tube–pistil interaction, specifically on the mechanisms of protein secretion and the identity of secreted factors that render pollen tubes competent for ovule-targeting, pollen tube reception, and fertilization. We used a high-throughput gel-free and label-free workflow utilizing nanoscale liquid chromatography (LC) and tandem mass spectrometry (MS/MS) to identify proteins of the *Nicotiana tabacum* semi-in vivo pollen tube secretome (SIV-PS). We observed an unprecedented bias towards unconventional protein secretion by the pollen tube. This type of secretion could be partly mediated by secreted nanovesicles (exosomes), suggesting for the first time a possible mechanism for long distance signaling by the pollen tube. Further, our results revealed a critical role for the endoplasmic reticulum (ER)–trans-Golgi network (TGN)–plasma membrane secretory components YIP4a,b and ECHIDNA on protein secretion, pollen tube growth, and competence of pollen tube targeting to the ovule, as well as fertilization. We show that this pathway can be hijacked by unconventionally secreted pollen tube proteins such as Translationally controlled tumor protein (TCTP), which could eventually be secreted to the extracellular matrix via exosomes and play a critical role in pollen tube–pistil signaling, fertilization, and seed production.

## Results

### A SIV method for high-throughput detection of pollen tube-secreted proteins following penetration through the pistil

Previously, we optimized a technique for tobacco pollen tube growth through the female stylar explant to detect pollen tube-secreted proteins following penetration through the female reproductive tissues. We termed this technique the semi-in vivo pollen tube-secretome (SIV-PS) assay [14]. In this study, we have coupled the SIV-PS assay with a gel-free and label-free semi-quantitative LC-MS/MS workflow to detect and quantify pollen tube-secreted proteins using minimum quantities of 2 μg of the pollen tube total secretome (Additional file 1: Figure S1). Our gel- and label-free strategy maximized detection of naturally low-abundant small secreted proteins by more than four orders of magnitude (relative to in-gel protein detection) and enabled us to detect proteins at concentrations as low as 0.45 parts per million (ppm), demonstrating the feasibility of the SIV-PS technique coupled with gel-free LC-MS/MS. Using the SIV-PS method, we have observed remarkable reproducibility with regard to pollen tube physiology, including growth, viability and intactness, cytoplasmic streaming, uniform tip morphology, callose wall and callose plug formation, and sperm cell production (Additional file 1: Figure S1). To test the purity of the secretome samples, we used alcohol dehydrogenase (ADH) as the most abundant cytosolic protein and performed an ADH assay to estimate cytosolic contamination (Additional file 1: Figure S1 and Additional file 2: Figure S2). Our results show that SIV-PS samples are pure with minimal cytosolic contamination relative to whole protein extracts from pollen tubes (15-fold less ADH activity in SIV-PS samples relative to control). The SIV-PS modifications maximized the identification of pistil-dependent pollen tube-secreted proteins induced following crosstalk with the female reproductive tissues compared with those secreted by in vitro germinated pollen tubes.

### Quantification of pollen tube-secreted proteins

To detect pollen tube-secreted proteins, we used label-free LC-MS/MS on two control samples (excised, unpollinated pistils, C1 and C2), four SIV-PS samples (SIV-PS1–4), four in vitro germinated pollen tube secretome samples after 24 h growth (PT24-PS1-4), four semi-in vivo whole proteome (SIV-PP1-4) samples, and four in vitro germinated pollen tube whole proteome samples (PT24-PP1-4). We used the Top3 label-free algorithm [17, 18] to determine approximate relative and absolute protein abundances from LC-MS/MS data, which allowed categorization of true secreted proteins over false positives for the SIV-PS samples as well as comparison of secretion dynamics across sample replicates and sample types. We identified an average of 1003 (2916 protein accessions) and 339 (1173 protein accessions) protein groups in individual SIV-PS and control samples, respectively. Protein relative quantification using the Top3 algorithm (up-regulated threefold or more and up-regulated in at least two SIV-PS samples and at the same time identified using three or more peptides) showed an average of 341 protein groups (801 protein accessions) to be likely pollen tube-secreted proteins following penetration through stigma and style (Additional file 3: Table S1). Our results show that quantitative LC-MS/MS can distinguish between secreted proteins and false negatives, particularly in cases where the same protein accessions were also detected in control unpollinated pistils. We provide three examples to demonstrate the resolution of our quantitative data with regard to determining true protein secretion (Additional file 4: Figure S3).

**Fig. 1:**
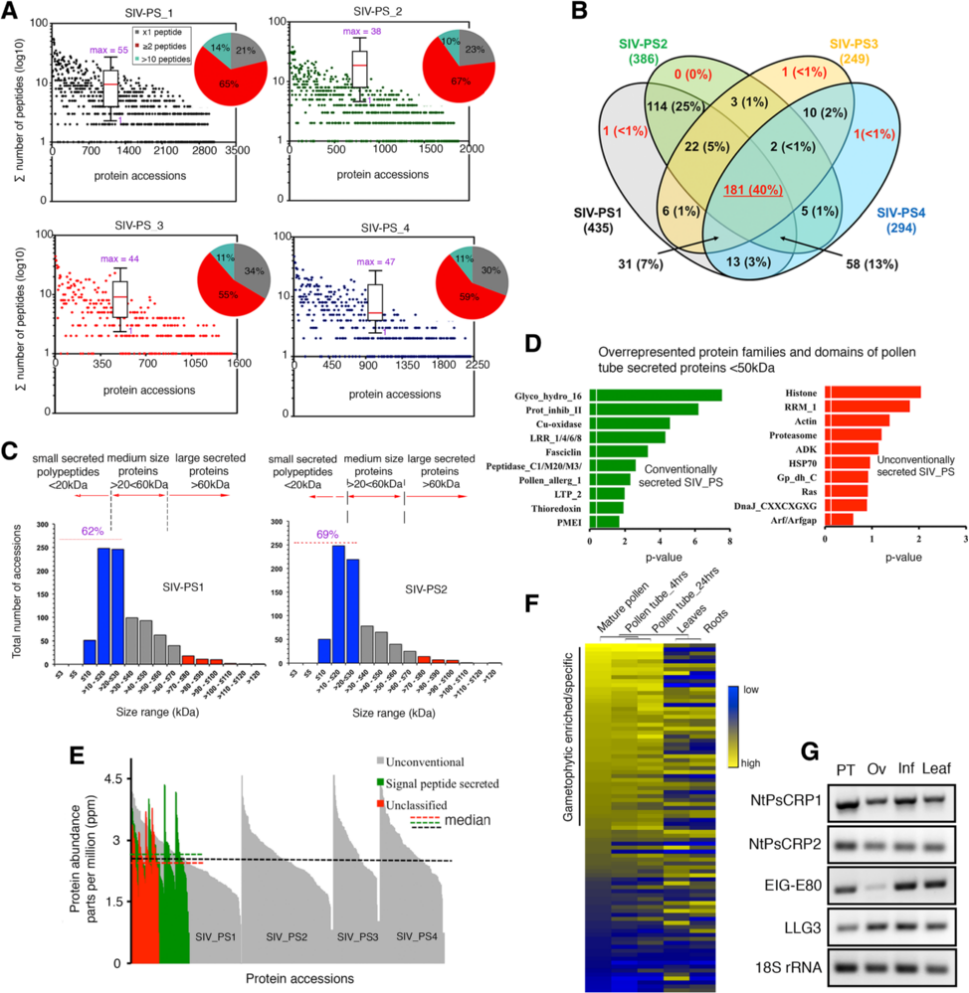
Label-free, high-throughput LC-MS/MS quantification of the SIV pollen tube secretome. **a** Numbers of peptides identified by LC-MS/MS. Box plots summarize peptide number distribution per protein accession within a single sample replicate with the median value designated by the *solid red line*. Appended pie charts show the distribution of peptide counts in arbitrary bins. **b** SIV-PS samples showing the reproducibility and dynamics of the secreted protein groups (threefold or more abundant in at least two SIV-PS samples relative to unpollinated controls and identified by at least three or more peptides). **c** Size distribution of pollen tube-secreted proteins, showing predominant bias towards small secreted proteins. SIV-PS1–2 are used as representatives of all four replicates. **d** In silico analysis of pollen tube-secreted proteins using SMART and Pfam databases of overrepresented protein families and domains. The v*ertical white line* indicates significance cutoff (*p* < 0.05). **e** Classified pollen tube-secreted proteins based on the Top3 algorithm showing the dominant presence of unconventionally secreted proteins, although these have comparable protein abundance to conventionally secreted proteins. **f** Heatmap derived from the Agilent tobacco microarray [19, 20] of transcripts encoding identified pollen tube-secreted proteins showing predominantly gametophytic enrichment. **g** Validated expression profile by semi-quantitative RT-PCR of selected pollen tube-secreted proteins assessed in this study. All samples analyzed were from *N. tabacum. NtPsCRP1/2 Nicotiana tabacum* cysteine-rich polypeptide protein 1 and 2, *EIG-E80* elicitor inducible gene subE80, *LLG3* Lorelei-like GPI-anchored protein 3, *PT* semi in vivo pollen tubes, *Ov* unfertilized ovules, *Inf* inflorescence

Raw peptide counts revealed that protein identification was predominantly based on two to ten peptides (65 %), followed by singletons (20 %) and those with more than ten total peptide counts (15 %) (Fig. 1a). Comparison of pollen tube secretome replicates based on absolute quantification of protein abundances in parts per million (see “Methods”) revealed that pollen tube protein secretion is consistent but also relatively dynamic, as observed from limited overlap of protein accessions between SIV-PS sample replicates (Fig. 1b). Protein size distribution showed a high frequency of secreted proteins of ≤20 kDa (Fig. 1c and Additional file 5: Table S2). Classification of protein families and domains highlighted glycoside-hydrolase family 16, proteinase inhibitor II, Cu-oxidase, LRR 1/4/6/8, and fasciclin as among the most overrepresented conventionally secreted proteins (Fig. 1d and Additional file 6: Table S3). In the unconventionally secreted protein group, histone, RNA-binding (RRM_1), HSP70, and proteasome families were the most frequent activities (Fig. 1d and Additional file 6: Table S3). Using the Top3 algorithm, the absolute abundance of pollen tube-secreted proteins was comparable regardless of the pathway of secretion utilized (Fig. 1e). Mapping of the identified pollen tube-secreted proteins to a tobacco microarray [19, 20] revealed a significant enrichment in gametophytic expression and, in some instances, specificity to the gametophyte (Fig. 1f). We conclude that: (1) the sensitivity of the gel-free sample preparation coupled with label-free LC-MS/MS analysis readily allowed quantitative evaluation and determination of pollen tube-secreted protein abundances; and (2) the pollen tube secretome is dominated by small secreted proteins with elevated hydrolase activities.

### The secretome of pollen tubes grown through the pistil is unique from that of in vitro cultured pollen tubes

**Fig. 2:**
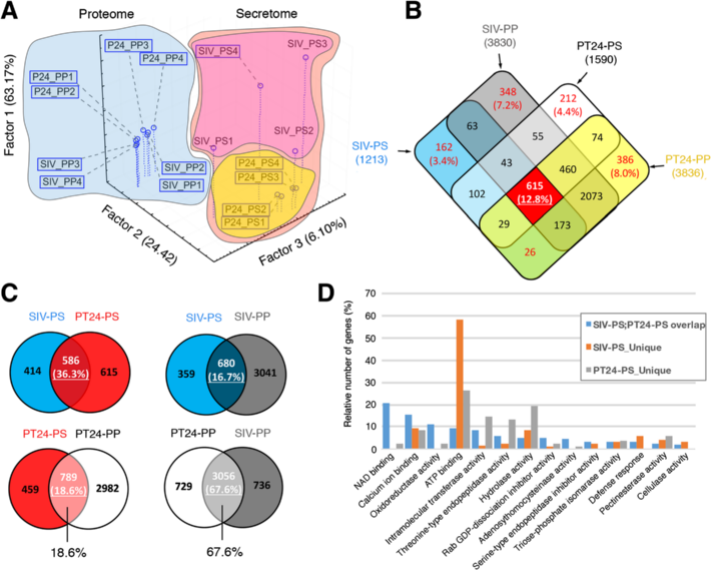
The secretome of pollen tubes grown through the pistil is unique from that of in vitro germinated pollen tubes and differs significantly from their respective proteomes. All sample types used for comparisons are representative of four replicates. **a** Three-dimensional principle component analysis of pollen tube secretomes (*PS*) and pollen tube proteomes (*PP*) from semi-in vivo (*SIV*) and 24 h in vitro germinated (*PT24*) pollen tubes. **b** Four-way Venn diagram of all sample types showing limited overlap between secretomes and proteomes but a relatively closer overlap between proteomes of SIV and in vitro germinated pollen tubes. **c** Pairwise comparison of sample similarities demonstrates that the secretome of pollen tubes grown through the pistil is unique from that of in vitro germinated pollen tubes and the pollen tube secretome is different from their respective pollen tube total proteomes, suggesting that regulated exo- and endocytosis occurs during pollen tube protein secretion. **d** GO term comparison of SIV and in vitro pollen tube secretomes, highlighting enriched activities where they overlap and protein accessions unique between the two sample types

To establish the unique physiology of the SIV pollen tube secretome following growth through the pistil, we compared it (SIV-PS) and the SIV proteome (SIV-PP) with the 24 h in vitro pollen tube secretome (PT24-PS) and its proteome (PT24-PP). Three-dimensional principal component analysis distinctively separated the samples into two groups, “secretome” and “proteome”, and spatially sub-grouped them further into “semi in vivo” and “in vitro” (Fig. 2a). These results clearly demonstrate that the pollen tube secretome is distinct from its proteome and that the SIV-PS is unique compared with PT24-PS. Comparison of protein supergroups (see materials and methods) from each sample type further emphasized the limited overlap between SIV and in vitro sample types (Fig. 2b). Pairwise comparison between the SIV-PS and PT24-PS using protein supergroups that constitute only accessions identified with three or more peptides and present in at least one replicate identified 414 protein supergroups that are unique to the SIV-PS (Fig. 2c, Additional file 7: Table S4). We observed only 36.3 % (586 protein groups) overlap between the SIV-PS and PT24-PS (Fig. 2c). Gene Ontology (GO) analysis revealed that the most overrepresented GO categories in the SIV-PS included ATP binding, defense response, and cellulase activities (Fig. 2c), all of which have been previously implicated in pollen tube growth and fertilization [4, 6]. The unique set of SIV-PS proteins secreted by pollen tubes grown through the pistil (Additional file 7: Table S4) represent some of the potential factors responsible for pollen tube guidance and ovule-targeting competence. They are candidates for further understanding how the pollen tube communicates with and is guided through the female reproductive tissues to achieve successful double fertilization.

Quantitative analysis of protein groups revealed that, despite the pollen tube secretome being dominated by unconventionally secreted proteins, both conventionally and unconventionally secreted proteins were secreted at comparable abundances (Fig. 1f). To establish whether the presence of a large number of unconventionally secreted proteins in the SIV-PS is a result of regulated secretion or is a consequence of a non-selective extrusion of cytoplasmic proteins due to extremely dynamic exocytosis and endocytosis in pollen tubes, a phenomena observed in tip growing cell types (Additional file 2: Figure S2) [21, 22], we compared the SIV-PS and PT24-PS samples with their respective total proteomes. Our results showed a limited overlap (16.7 % and 18.6 %, respectively) between the secretome and total proteome, suggesting that the observed dominant unconventional protein secretion of the SIV-PS is a result of a regulated process and not a consequence of extreme exocytosis and endocytosis (Fig. 2b, c).

More interestingly, the pollen tube proteomes from SIV and in vitro grown pollen tubes showed a much bigger overlap (67.6 %) than their respective secretomes (Fig. 2c). These results suggest that distinct pistil factors (for instance, the female-secreted pollen tube guidance signals) might affect the pollen tube secretome independently of other pistil factors that influence the SIV pollen tube proteome or transcriptome.

### Correlation of the tobacco SIV-PS with the *Arabidopsis* SIV transcriptome

To understand tobacco pollen tube secretion dynamics, we studied the correlation between the tobacco SIV-PS and transcript profiles from *Arabidopsis* SIV microarray data [15]. Mapping of a subset of the tobacco pollen tube-secreted proteins (only those identified in the UniProt database) to close homologs from the *Arabidopsis* SIV pollen tube transcriptome revealed an astonishing 90.65 % (681/739) overlap, of which 372 genes (50.34 %) showed reliable expression in all three microarray replicates (Additional file 8: Table S5). These results suggest that the 681 genes expressed in *Arabidopsis* SIV pollen tubes are also detected in the tobacco SIV-PS. The near-complete overlap between the two datasets also suggests that the physiological response of pollen tubes following growth through the stigma and style is strongly conserved at the transcriptome level as well as at the secretome level in both plant species.

**Fig. 3:**
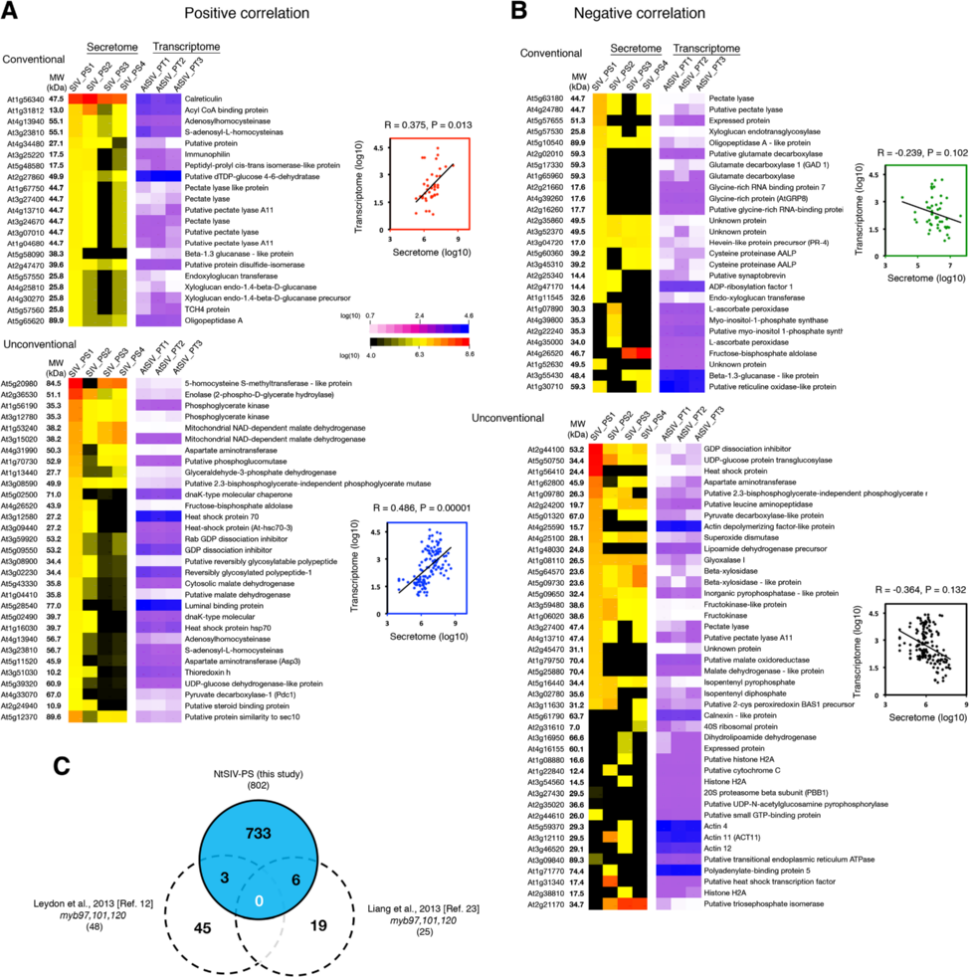
Semi-in vivo transcriptome–secretome correlation. Pollen tube-secreted proteins showed moderate positive (**a**) and negative (**b**) correlation, but not linear correlation, with transcripts of the *Arabidopsis* SIV pollen tube transcriptome [15]. To the *right* of each heatmap are scatter plots with Pearson correlation coefficient scores and confidence values at *p*< 0.01. **c** Comparison of tobacco SIV-PS with downstream targets of MYB97, 101, and 120 identified in [12, 23]

When we compared transcript profiles with the corresponding secreted protein abundances, we found that, for the bulk of the dataset, the pollen tube protein secretion was uncoupled from gene expression profiles (Fig. 3). The relative abundances of secreted proteins showed no linear correlations with the transcript levels, although we did observe moderate positive (R1 = 0.375, *p* = 0.013; R2 = 0.486, *p* = 0.00001) and negative (R1 = −0.239, *p* = 0.102; R2 = −0.364, *p* = 0.132) correlations based on Pearson correlation coefficient scores (Fig. 3a, b). These data suggest that the pollen tube secretome is a specialized subset of the male gametophyte signaling repertoire and cannot be predicted from transcriptional profiling alone.

Moreover, two previous studies by Leydon et al. [12] and Liang et al. [23] have shown that simultaneous knockdown of pollen-expressed MYB transcription factors (*MYB97*, *MYB101*, and *MYB120*) in SIV pollen tubes led to the down-regulation of target genes, some of which were essential for pollen tube guidance, reception, and fertility [12, 23]. Comparison of these MYB97/101/120 targets, 48 genes from Leydon et al. and 25 genes from Liang et al., with the tobacco SIV-PS identified overlaps of 3/48 (6 %) and 6/25 (24 %), respectively (Fig. 3c). These overlapping proteins include galactokinase 2 (ATGALK2), cellulase 3 (CEL3) with hydrolase activity, and myo-inositol-1-phosphate synthase 2 (MIPS) from the Leydon et al. study and CC (carboxylate clamp)-TPR (tetratricopeptide repeat) protein (AT5G48570), similar to endo-xyloglucan transferase (AT4G30270), histone H2A protein (AT1G51060), HSP70B (HEAT SHOCK PROTEIN 70B; AT1G16030), HSP20-like chaperones superfamily protein (AT1G07400) and HSP70 (DG4; AT3G12580) from the Liang et al. study. The minimum overlap observed between the tobacco SIV-PS and potential downstream targets of MYB97/101/120 transcription factors could be due to tobacco genes encoding pollen tube-secreted proteins not being predominant targets of the *MYB97;MYB101;MYB120* transcription unit. This implies possible activities of multiple transcriptional pathways are involved in coordinating pollen tube-secreted protein transcriptional activation in tobacco. It is also possible that MYB97/101/120 transcriptional regulation of pollen tube-secreted proteins could be species-specific. The lack of positive correlation between secreted protein abundance and their corresponding transcripts could imply that transcriptional activation and protein secretion are regulated independently, which might influence protein/transcript detection and hence lack of correlation.

Further, a comparison with the protein families of the MYB97/101/120 downstream target genes classified as potentially secreted proteins [12] identified that three-quarters of them were in common with the tobacco SIV-PS. These include alpha carbonic anhydrase (in tobacco we identified beta carbonic anhydrase, Nt_000566), thionin (in tobacco cysteine-rich family proteins Nt_017951, *Q9SDN7-NtPsCRP2* (characterized in this study), Nt_013502) and GDSL-motif lipase/hydrolase (in tobacco we also identified GDSL-motif lipase/hydrolases Nt_021542, Nt_066702, Nt_015437, and Nt_001237). We did not detect a self-incompatibility (SI)-related protein identified in *Arabidopsis* but instead we identified two highly abundant secreted RNases (discussed in section "SP-containing proteins secreted following pollen tube– pistil interaction) that are also involved in self-incompatibility during pollen tube growth through the pistil. The identification of these common protein families between the tobacco pollen tube secretome and the downstream targets of *Arabidopsis* MYB97/101/120 in SIV pollen tubes suggests that pollen tube perception of female guidance signals, recognition of pollen tube arrival by the female, and the ability of pollen tubes to respond to these signals is likely conserved in both species.

### In silico classification of pollen tube-secreted proteins and their post-translational modifications

Conventionally secreted proteins contain a short N-terminal signal peptide (SP) [24] and are secreted through the ER–Golgi–TGN pathway. In silico analysis using the SignalP-TM prediction database [25] of protein groups with three or more peptides and up-regulated by threefold or more relative to the median of the two control samples, revealed that 15.69 % of the pollen tube-secreted proteins possessed an N-terminal SP (Additional file 9: Figure S4a). Analysis using the SecretomeP server [26] further supported that > 85 % of the SIV secretome likely constitutes true secreted proteins (Additional file 9: Figure S4a). To rule out their possible retention on the ER membrane system, we manually scanned for the presence of ER-retention motifs (HDEL/KDEL) using ProSite scan [27]. None of the accessions were found to possess likely functional HDEL/KDEL core motifs, signifying their secretion towards the plasma membrane and the extracellular matrix. Analysis of the remaining non-SP-containing proteins intriguingly highlighted > 57.18 % as potential unconventionally secreted pollen tube proteins (Additional file 9: Figure S4a) [28]. This high proportion of unconventionally secreted proteins suggests that the unconventional protein secretion pathways could be the predominant mechanism for pollen tube protein secretion.

Next, we assessed the possibility of post-translational modifications of the identified secreted proteins. ER–Golgi–TGN-secreted proteins are known to commonly undergo post-translational N-linked glycosylation within the tripeptide Asp-Xaa-Ser/Thr sequon [29]. Using the NetNGlyc v1.0 [29] and GPP [30] databases, we revealed that 81.33 % of the conventionally secreted proteins and, unexpectedly, 41.30 % of the unconventionally secreted proteins were predicted to be N-glycosylated (Additional file 9: Figure S4a). We confirmed glycosylation of pollen tube secreted proteins by concavalin A glycoprotein staining, which demonstrated that a subset of the pollen tube secretome underwent N-glycosylation (Additional file 9: Figure S4e).

We then investigated post-translational palmitoylation of the pollen tube-secreted proteins. Palmitoylation is the covalent attachment of palmitic acid, most frequently at a cysteine residue, to enhance protein hydrophobicity and affinity for the plasma membrane. Interestingly, 71.76 % of conventionally and 40.12 % of unconventionally secreted proteins were predicted to be palmitoylated (Additional file 9: Figure S4a). Additionally, 14.75 % of the conventionally secreted proteins were predicted to be post-translationally modified at the C-terminal ω-site for glycophosphatidylinositol (GPI)-anchoring (Additional file 9: Figure S4b). Surprisingly, the majority (61 %) of the predicted GPI-anchored proteins (GAPs) were also predicted to undergo palmitoylation. One possible explanation for this unexpected observation is that a subset of proteins destined to the plasma membrane but lack transmembrane helices (TMHs) could be palmitoylated to enhance their affinity for the lateral plasma membrane and thereafter anchored to the plasma membrane potentially with the GPI motif. We therefore investigated whether pollen tube-secreted proteins possess class I or class II TMHs. Using TMHMM v2.0 algorithms, our analysis revealed no reliable prediction for the presence of TMHs in both conventionally (average of 11.03 amino acids in TMHs) and unconventionally secreted proteins (average of 1.23 amino acids in TMHs) (Additional file 10: Figure S5a). In comparison, TMH prediction with known pollen tube plasma membrane signaling proteins (ACA9, PRK2, PRK4, ANX1, and ANX2) revealed an average score of 70.49, verifying confidence in the algorithms used for prediction of TMHs (Additional file 10: Figure S5a). A similar test with LIP1/2 (Loss in pollen tube guidance 1/2 receptor kinases) [31], which localizes to the pollen tube plasma membrane only through palmitoylation, showed an average score of zero (Additional file 10: Figure S5a). We concluded that pollen tube-secreted proteins are less likely to posses TMHs; instead, putative pollen tube receptor proteins could function through transient anchors on the plasma membrane following post-translational modification and secretion.

### Subcellular localization of pollen tube-secreted proteins

We used LocTree2 prediction algorithms with a pre-computed kernel matrix in support vector machine learning (SVM) and implemented tree-like hierarchy subcellular protein sorting to assess the subcellular localization of the pollen tube-secreted proteins [32]. Approximately 68.5 % of SP-containing proteins were classified as secreted and a further 16.5 % as localizing in secretory compartments, including the ER lumen, Golgi apparatus, vacuole, and plasma membrane, accounting for a total of 85 % as secreted with a reliability index score of 60.1 (Additional file 9: Figure S4c). Intriguingly, LocTree2 predicted 13.7 % of the unconventionally secreted proteins as secreted and an additional 7.1 % that were destined to be in or transported through secretory compartments (Additional file 9: Figure S4c). The majority of the unconventionally secreted proteins (> 42.9 %) were predicted to localize to the cytoplasm (Additional file 9: Figure S4). However, the term “cytoplasm” was secondarily associated with GO terms secretory granules, extracellular space, Golgi intermediate, membrane bound vesicles, mitochondrion, nucleus, and protein complexes, among others. Independent analysis using TargetP1.1 [33] showed that over 94 % of the SP-containing proteins were predicted as secreted (*p* > 0.95; Additional file 10: Figure S5b). Only 6.5 % of unconventionally secreted proteins were classified as secreted. Nonetheless, 58.5 % of the unconventionally secreted proteins were classified as “ambiguous localization” (Additional file 10: Figure S5b). The “ambiguous” domain is derived from the lack of significant differences between the specificity scores for different subcellular compartments [33]. Therefore, our observation suggests that the identified unconventionally secreted proteins are less likely to be truly retained cytosolic proteins; instead, they could utilize as yet unknown features for their secretion.

Analysis of enriched GO terms associated with the predicted subcellular localization revealed predominant protein secretion by the pollen tube to the extracellular space and less frequently protein retention in secretory pathways, the ER, and vacuoles (Additional file 10: Figure S5c). These results support our earlier observations that the identified secreted proteins lack ER-retention signals and transmembrane domains (Additional file 10: Figure S5a).

GO-slim term enrichment analysis revealed the terms signaling, post-translational protein modification, response to stimulus, small molecule metabolic process, GTPase activity, calcium and copper ion binding, endopeptidase activity, hydrolase and redox activities, as well as intracellular membrane transport to be enriched in the pollen tube secretome (Additional file 11: Figure S6a). Interestingly, the categories “proteins anchored to membrane” and “serine-type endopeptidase inhibitor activity” were the most enriched in conventionally secreted proteins, whereas defense response, L-ascorbate peroxidase activity, cell redox homeostasis, and calcium ion binding were the most enriched terms of the unconventionally secreted protein subgroup. A full list of pollen tube secretome enriched GO terms is presented in Additional file 8: Table S5.

### Identification of predicted palmitoylated and GPI-anchored secreted proteins as potential pollen tube transient receptors

We searched our pollen tube secretome for putative pollen tube-secreted receptors. Since secreted proteins should not possess transmembrane helices, we searched for secreted proteins predicted to be palmitoylated and those with a plasma membrane GPI anchor and ω-site with a cutoff false discovery rate (FDR) of 0.1 %; 14.75 % were predicted to be bona fide GAPs (Additional file 9: Figure S4b). Among the reliably predicted secreted GAPs were LORELEI-like GPI anchored protein 3 (LLG3, 97 ppm), lipid transfer protein (18 kDa LTP, Nt_005942, 4590 ppm), a 24 kDa unknown protein (Nt_002352, 24 ppm), 19 kDa nsGRP-2 (Nt_058890, 198 ppm), 29 kDa plasmodesmata callose-binding protein 3 (29 kDa PDCB3, Nt_004725, 258 ppm), 26 kDa glycosyl hydrolase family 17 protein (Nt_031990, 954 ppm), glycosylphosphatidylinositol-anchored lipid protein transfer 1 (LTPG1, Nt_003387-2, 397 ppm) and 20 kDa NtEPc-like protein (Nt_051987, 578 ppm). Notably, NtEPa–c were previously purified as markers for the embryogenic dedifferentiation of immature tobacco pollen grains cultivated in vitro [34].

Intriguingly, we observed that 61.7 % of the predicted GAPs were also predicted to be palmitoylated. The palmitoylation could increase affinity for the plasma membrane and the GPI modification is likely to mediate anchoring to the outer face of the plasma membrane. We cautiously propose that some of these predicted palmitoylated GAPs are potential pollen tube transient receptors that could facilitate signal perception by binding ligands secreted by female reproductive tissues.

### Pollen tube-secreted kinases and cysteine-rich proteins as putative carbohydrate modifiers and signal transducers

We searched for kinases among the pollen tube-secreted proteins as putative modifiers of carbohydrates in the extracellular matrix. Modified carbohydrates could function as ligands for some protein receptors. The most abundant kinases identified were phosphoglycerate kinases, hexokinases, fructokinases, adenylate kinases, and pyruvate kinases. Three phosphoglycerate kinases, Q42962 (2354 ppm), Nt_011032 (2130 ppm) and Nt_008716 (1948 ppm) containing the phosphoglycerate kinase domain at the C-terminus were predicted to be secreted through the unconventional secretion pathway. Other kinases included a PfkB-type carbohydrate kinase family protein (Nt_054946, 498 ppm), UMP-CMP cytidylate kinase (PYR6; Nt_001852; 497 ppm), SHV3-like 4 glycerolphosphodiester kinase (Nt_053416, 162 ppm) and adenosine kinase (Nt_046116, 24 ppm). The PfkB kinase contains the PfkB domain and ribokinase domain whereas PYR6 contains a P-loop NTPase domain and cytidylate kinase domain.

Cysteine-rich proteins (CRPs) have also been discovered to function as ligands during signal transduction. Some of the CRPs detected in SIV-PS samples included OTU-like cysteine protease family protein (Nt_006421, 104 ppm), cysteine-rich proteins (Nt_017951, 396 ppm and Nt_013502, 57 ppm), NRCL4 (Nt_029302, 76 ppm), and a cysteine proteinase inhibitor (Nt_051779, 97 ppm). The full list is searchable in Additional file 3: Table S1.

### SP-containing proteins secreted following pollen tube–pistil interaction

Approximately 16 % of the pollen tube secretome is comprised of SP-containing proteins (Additional file 9: Figure S4a). These include two highly abundant RNases belonging to the RNase T2 family. The T2 family S-RNases are female determinants of self-incompatibility in the Solanaceae, Rosaceae, and Scrophulariaceae, whereas S-locus F-box (SLF)/S-haplotype-specific F-box proteins are male determinants of self-incompatibility within these families [35]. Pollen tube-secreted RNase NE (Q40382, 234 ppm) and RNase1 (Q9MB71, 317 ppm) were both detected in both pollinated and unpollinated pistils but convincingly absent in SIV pollen tube total proteomes. More interestingly, both RNases were not present in the in vitro pollen tube secretome or total pollen tube proteomes (Additional file 3: Table S1 and Additional file 7: Table S4). NtRNase1 shares 99.53 % identity with *Nicotiana tomentosiformis* extracellular ribonuclease LE-like (XM_009627901.1) and RNase NE shares 83.5 % identity with *Solanum lycopersicum* extracellular ribonuclease LE of the T2 family. Moreover, NtRNase1 and RNase EN show 62.3 % similarity with PD1, an S-like ribonuclease from *Prunus dulcis* (Additional file 12: Figure S7a). To establish the origin of the detected pollen tube-secreted RNases, we performed semi-quantitative RT-PCR analysis and observed that both NtRNase1 and RNase EN were specifically expressed in unpollinated and pollinated stigmas and style of *N. tabacum* (14 h post-pollination) but absent in in vitro grown pollen tubes 24 h after germination (Additional file 12: Figure S7b). Since *N. tabacum* is a self-compatible species, our results indicate that pollen tubes grown through the pistil uptake secreted RNases [36, 37] from transmitting tract extracellular matrix and secrete them out in cases of matching S-allele haplotypes, supporting the inhibitory model of self-compatibility/incompatibility [36, 37].

Another conventionally secreted protein identified was 11.7 kDa tobacco cysteine-rich defensin-like protein, here annotated as *N. tabacum* Pollen tube-secreted cysteine rich protein 1 (NtPsCRP1). NtPsCRP1 belongs to the subgroup of the DEFL family with CSαβ and γ-core motifs, similar to TfCRP1 (LURE1), TfCRP2, and TfCRP3 (LURE 2) identified in *T. fournieri* [3]. NtPsCRP1 is closely related to TfCRP1 and TfCRP3, with eight conserved cysteine residues and an N-terminal signal peptide (Additional file 12: Figure S7c, d). Moreover, NtPsCRP1 was only detected in the SIV-PS samples and not in unpollinated pistil controls. Further, NtPsCRP1 expression is enriched in SIV pollen tubes but also occurs in unfertilized ovules as verified by semi-quantitative RT-PCR analysis (Fig. 1g). We also detected secretion of another CRP of 8.3 kDa (Q9SDN7, 295 ppm) that possessed six conserved cysteine residues in its mature form and was specifically secreted in pollinated pistils. It belongs to the pollen allergen Ole-e-6 superfamily and has 98.6 % homology to NtP-CysR, a *N. tabacum* pollen CRP corresponding to a 63 amino acid secreted protein precursor enriched in olive pollen [38]. We annotated this protein as *N. tabacum* Pollen tube-secreted cysteine rich protein 2 (NtPsCRP2). Semi-quantitative RT-PCR analysis revealed a slightly higher expression of NtPsCRP2 in pollen tubes relative to unfertilized ovules (Fig. 1g).

Other conventionally secreted proteins detected were enzymes involved in carbohydrate metabolism predicted to be involved in cell wall modification. These include beta-expansin-like protein (Nt_065866 422 ppm), pectinesterase (K4AWN9, 1084 ppm), and P18 with putative pectinesterase activity (O65849, 1769 ppm). Detected cell wall proteoglycans, arabinogalactan proteins (AGPs), included fasciclin-like arabinogalactan protein 14 (FLA14; Nt_005615; 3478 ppm), FLA3 (Nt_016813, 3115 ppm), FLA2 (Nt_002342, 116 ppm), FLA8 (Nt_002342, 257 ppm), and UDP-arabinopyranose mutase 2-like (UPI00032A57BC, 590 ppm) as unconventionally secreted proteins. Several extensin-like proteins were also identified, including pollen-specific leucine-repeats extensin-like protein (UPI0002339BC6, 155 ppm) and 120 kDa pistil-extensin-like proteins (PELP, Q49I34, 115 ppm). Pistils-extensin-like proteins were classified as major components of the transmitting tract extracellular matrix [39, 40]. PELPIII was recently demonstrated to be essential for interspecific incompatibility in *N. tabacum* by inhibiting interspecific pollen tube growth [41], whereas Pex1 was identified as a pollen-specific extensin in *Zea mays* that is secreted and glycosylated [42] and functions as a male factor in pollen tube growth through the transmitting tract [43].

Conventionally secreted proteins predicted to function in pollen tube guidance and directional growth were also detected, in particular members of the plant lipid transfer protein (LTP) family. These include non-specific lipid-transfer proteins NtLTP1 (Q42952, 660 ppm), NtLTP3 (F2ZAM0, 142 ppm), and NtLTP5 (Q6E0U9, 142 ppm) (Additional file 3: Table S1). Plant LTPs and LTP-like proteins were implicated in diverse extracellular functions, including anti-fungal and anti-microbial activities, cuticular wax deposition, and cell wall loosening [44]. We have also detected lipid-binding proteins such as the annexin calcium-dependent phospholipid binding protein AN2 (Nt_004158, 134 ppm). Other novel conventionally secreted proteins with potential roles as pollen tube-guidance proteins included luminal binding protein 5 (Q03685, 525 ppm), peptidyl-prolyl cis-trans isomerase (B2BF99, 227 ppm), endoplasmic reticulum HSC70-cognate binding protein (685 ppm), proteinase inhibitor (Q84L56, 58 ppm), and tobacco NTS1 protein (Q9FV64, 1405 ppm).

### The LAT52-PRK2/PRK4 receptor kinase module could function late in pollen tube signal perception

We detected secretion of the CRP LAT52 (Nt_005952; 21394 ppm; a representative accession) from the Ole e I family in SIV-PS samples 48 h post-pollination and *Lb*LAT52-like small protein (H6VN37, 88 ppm) from *Lycium barbarum* (91 % identity) in the unpollinated pistil control (Additional file 3: Table S1 and Additional file 13: Figure S8e–g). The detection of *Lb*LAT52-like small protein in unpollinated pistils is likely due to peptide homology (detected as a single unique peptide that shared homology with LAT52 from *S. lycopersicum*) rather than true secretion from the pistil tissues. The interaction of LAT52 with its plasma membrane receptor kinases, the PRK2 and PRK4 ligand–receptor complex, is known as an essential endocrine module that promotes pollen germination and pollen tube growth [10]. LAT52 was detected in all four replicates of both SIV and in vitro pollen tube secretomes as well as in their respective total proteomes (Additional file 3: Table S1 and Additional file 7: Table S4). Contrarily, PRK2 and PRK4 were not detected in any of the secretome samples nor in the total proteome samples, verifying the high purity of the secretome samples. Lack of their detection in the total proteomes is likely due to a generic protocol used for total protein extraction rather than a membrane protein enrichment protocol. Analysis of their expression using microarray data and by RT-PCR revealed abundant expression of PRK2 and PRK4 receptor kinases specifically in mature pollen grains, in vitro cultivated pollen tubes after 4 h and 24 h, as well as SIV pollen tubes of *Arabidopsis* and tobacco (Additional file 13: Figure S8f,g). We could not detect expression of PRK2 or PRK4 in unfertilized ovules of *N. tabacum*, implying conserved male gametophyte-specific expression (Additional file 13: Figure S8g); both genes were also not expressed in the sporophyte (Additional file 13: Figure S8F,G). We propose that the detection of pollen tube-secreted LAT52 ligands and expression of PRK2/4 receptors (Additional file 13: Figure S8h) late during tobacco pollen tube growth suggests a likely continuous function of the LAT52–PRK2/4 complex module, either to quench or to establish homeostasis with PRK-RopGEF-promoted activities or to perform an as yet unknown role in male–female signal perception.

### Hindrance of the N-terminal SP impairs secretion of pollen tube EIG-E80 protein to the apoplast of leaf epidermal cells

**Fig. 4:**
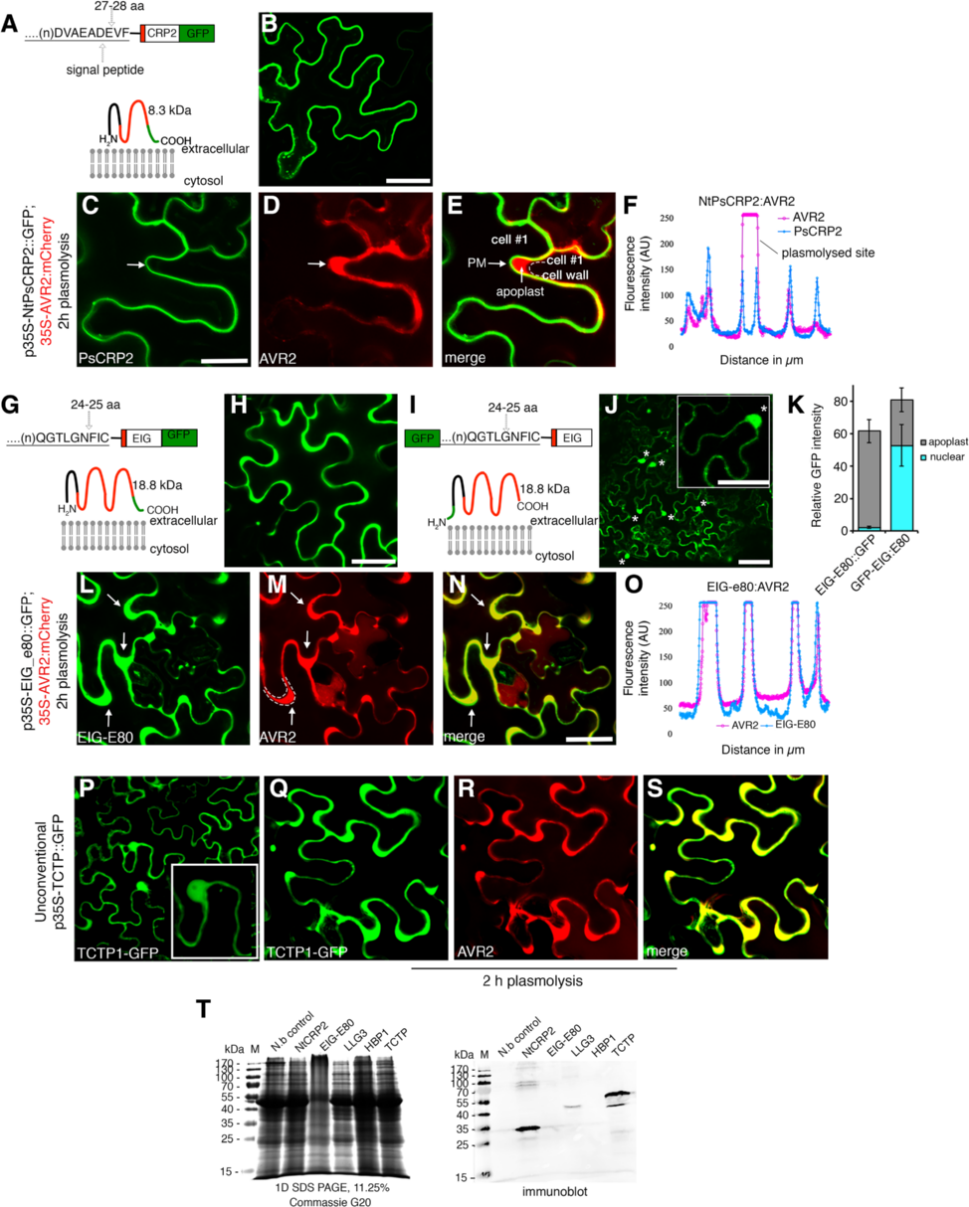
Interference of the N-terminal signal peptide compromises apoplastic localization of the EIG-E80 pollen tube-secreted protein. Secretion of pollen tube-secreted proteins to the proximity of the plasma membrane and the apoplast of tobacco leaf epidermal cells is signal peptide-dependent. **a, b** Chimeric construct of pollen tube-secreted cysteine-rich polypeptide protein 2 (NtPsCRP2) and its predicted topology showing extracellular localization. **c–e** Verification of NtPsCRP2 localization near the plasma membrane (*PM*) by plasmolysis of tobacco epidermal cells co-infiltrated with apoplastic viral AVR2-mCherry. The *arrow* shows plasmolyzed regions with detached plasma membrane and apoplastic localized PR1a-AVR2-mCherry. **f** Two-channel confocal laser scan profile during co-localization of NtPsCRP2 with apoplastic marker AVR2-mCherry following plasmolysis. *AU* arbitrary units. **g, h** Chimeric construct of elicitor-induced protein E80 (EIG-E80) and its predicted topology showing apoplastic/extracellular localization. **i, j** Reconstituted GFP:EIG-E80 chimeric construct with blocked signal peptide resulted in partial apoplastic localization and predominant nuclear localization instead. **k** Subcellular quantification of the modified GFP:EIG-E80 expression. Error bars represent ± standard deviation. **l–o** Verification of EIG-E80:GFP apoplastic localization by plasmolysis with viral AVR2 apoplastic marker. *Arrows* indicate plasmolysed regions. **p** Localization of the unconventionally secreted tobacco pollen tube protein Translationally controlled tumor protein (TCTP) showing nucleoplasm, cytosol, and apoplastic localization. **q–s** Verification of TCTP apoplastic localization. **t** Immunodetection verification of ectopically expressed GFP chimeric pollen tube-secreted proteins with rat anti-GFP monoclonal antibodies in tobacco epidermal cells. Commassie G250 stain of total protein extract (*left*) and immunoblot (*right*) 48 h post-infiltration. Scale bars = 10 μM. *N.b* untransformed Nicotiana benthamiana

To demonstrate utilization of the N-terminal SP motif for the conventional secretion of pollen tube proteins in a heterologous system, we selected NtPsCRP2 and elicitor-induced protein E80 (EIG-E80) to assess their subcellular localization with a blocked N-terminal SP (Fig. 4). Transient expression in tobacco leaf epidermal cells revealed unambiguous secretion of NtPsCRP2 to the proximity of the plasma membrane and EIG-E80 to the apoplast (Fig. 4a, b, g, h). We verified their respective localizations by plasmolysis and co-expression of viral apoplastic protein AVR2-mCherry (Fig. 4c–f, l–o). We then restructured the EIG-E80 protein conformation by masking its N-terminal SP through C-terminal green fluorescent protein (GFP) fusion (Fig. 4i). Protein topology analysis predicted extracellular localization for both conformations of the EIG-E80 protein (Fig. 4g, i). Remarkably, the restructured GFP–EIG-E80 chimeric protein failed to be secreted effectively to the apoplast, instead predominantly accumulated into the nucleus (Fig. 4j, k). These results demonstrate the significance of the SP position in directing protein entry into the secretory pathway. A weak signal was still observed in the apoplast, suggesting partial secretion. The ectopic expression of selected pollen tube-secreted proteins in leaves of *Nicotiana benthamiana* was verified by western blot analysis (Fig. 4t). We concluded that these identified proteins posses functional SP and are likely to utilize the ER–TGN secretion pathway, providing indirect evidence for their secretion from the pollen tubes during a crosstalk with the female reproductive tissues.

### NtPsCRP2 is associated with dynamic Golgi-derived vesicles in close proximity of the plasma membrane

**Fig. 5:**
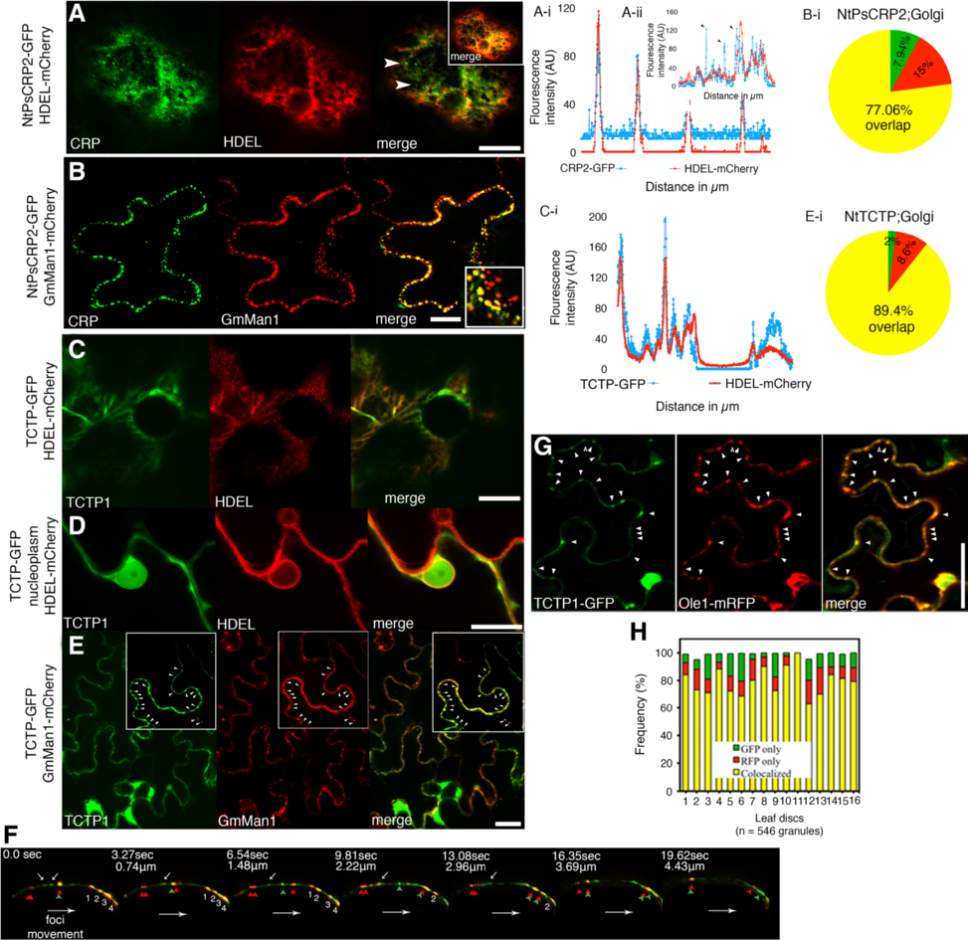
NtPsCRP2 and unconventionally secreted NtTCTP enter the ER and co-localize with the Golgi marker GmMan1 and exosome marker Ole1. **a** Confocal micrographs showing co-localization NtPsCRP2-GFP with the ER retention marker HDEL-mCherry with the exception of GFP foci (*white arrowheads*). Two-channel profile overlap (*a-i*). Emphasis on the lack of complete overlap (*black arrowheads*) of ER-HDEL with NtPsCRP2-GFP foci (*a-ii*). **b** NtPsCRP2 co-localization with the ER–Golgi vesicle marker GmMan1-mCherry from soybean. Frequency of co-localization (29 confocal slices, n = 340) (*b-i*). **c** Co-localization of unconventionally secreted NtTCTP with ER HDEL-mCherry. Visualization of ER co-localization of NtTCTP by two-channel pixel-intensity profiling (*c-i*). **d** NtTCTP also co-localizes with the ER–nuclear lamina and is present in the nucleoplasm. **e** NtTCTP co-localization with the Golgi vesicle marker GmMan1-mCherry. *Arrowheads* indicate granules formed by TCTP-GFP and those by GmMan1-mCherry. **e–i** Frequency of overlapping foci observed in 32 confocal slices (n = 292). **f** Dynamics of NtPsCRP2-GFP foci (*green*) and Golgi-derived vesicles (*red*) in the proximity of the plasma membrane. The *top white arrows* point to tethered NtPsCRP2-GFP foci over time, the *red arrowheads* Golgi vesicles alone, the *green arrowheads* NtPsCRP2-GFP foci alone, and the *red-green arrowheads* co-localized NtCRP1-Golgi signals, and foci *1–4* are additional NtCRP1-Golgi co-localized vesicles migrating clockwise towards the membrane horizon over time. Note the appearance and disappearance of co-localized NtPsCRP2-Golgi vesicles as well as NtPsCRP2-GFP foci alone. Scale bars = 20 μM. **g** NtTCTP co-localized with Ole1 in potential nanovesicle exosomes. Co-expression of NtTCTP-GFP with AtOle1-mRFP in tobacco leaf epidermal cells revealed granular co-localizations (marked with *arrowheads*). **h** Frequency of co-localizations observed from multiple leaf discs. Scale bars = 10 μM, *RFP* red fluorescent protein

The near-plasma membrane localization of NtPsCRP2-GFP in leaf epidermal cells prompted us to investigate the exact pathway for its secretion. We co-expressed NtPsCRP2-GFP in leaf epidermal cells with an ER retention marker, HDEL-mCherry, under the CaMV 35S promoter. NtPsCRP2-GFP distinctly decorated the ER lumen and co-localized with the HDEL-mCherry, supporting NtPsCRP2 ER localization (Fig. 5a, a-i). Additionally, NtPsCRP2-GFP formed GFP foci that did not co-localize with the ER retention marker (Fig. 5a a-ii, ). When we co-expressed NtPsCRP2-GFP with an early ER–Golgi marker, GmMan1-mCherry derived from soybean [45], a clear overlap of 77 % (*n* = 340 confocal slices) was observed, suggesting NtPsCRP2 is translocated from the ER to the Golgi and TGN (Fig. 5b, b-i). Furthermore, NtPsCRP2-GFP;GmMan1-mCherry-associated Golgi-derived vesicles showed stop-and-go, unidirectional, non-zigzag, cycling movements along the proximity of the plasma membrane (Fig. 5f). We observed dynamic GmMan1-labeled vesicles alone as well as NtPsCRP2-GFP-loaded GmMan1-mCherry vesicles (Fig. 5f). Our observations suggest a common route for NtPsCRP2 delivery via Golgi-derived vesicles to the plasma membrane and recycling of unloaded vesicles. We have identified similar NtPsCRP2 stop-and-go cycling movements in root epidermal cells of *Arabidopsis thaliana* (Additional file 14: Figure S9a, b, Additional file 15: Movie S1, and Additional file 16: Movie S2). The rationale for such NtPsCRP2-GFP;GmMan1-mCherry vesicle movements is still unclear, and whether actin and microtubule dynamics are involved in implementing such movements.

### Unconventionally secreted proteins are the dominant class of pollen tube-secreted proteins following pollen tube–pistil interaction

In our SIV-PS, 57 % of the secreted proteins identified were predicted to be unconventionally secreted proteins. Secreted proteins without the canonical N-terminal SP have been identified in animals and yeast but very few have been reported in plants [28, 46, 47]. In *Arabidopsis*, 18 % of its total proteome is predicted to be secreted and 40–70 % of the proteins identified in secretome studies do not contain the N-terminal SP [48]. We identified unconventionally secreted pollen tube proteins with sizes ranging between 5.4 and 138 kDa, with the most predominant class being 20–50 kDa (50.2 %), followed by proteins of < 20 kDa (24 %) (Additional file 3: Table S1, Additional file 8: Table S5, and Additional file 17: Table S6). In comparison, the size of conventionally secreted proteins ranged between 8.4 and 105 kDa and were also predominately 20–50 kDa (40.1 %) and those < 20 kDa (9.1 %) (Additional file 3: Table S1, Additional file 8: Table S5, and Additional file 17: Table S6). Some selected unconventionally secreted pollen tube proteins identified included glyceraldehyde 3-phosphate dehydrogenase (H9U034, 312 ppm), dihydrolipoyl dehydrogenase from *S. lycopersicum* (Q6QJL7, 2401 ppm), elicitor inducible (EIG-I24) tobacco gene (Q9FXS7, 235 ppm), ubiquitin fold-modifier (G7K8X5, 237 ppm), and pollen-specific actin-depolymerizing factor 1 (Q8H2B7, 104 ppm); for the complete list see Additional file 3: Table S1 and Additional file 8: Table S5. Our observations imply that unconventional secretion might be a default or dominant secretory pathway in growing pollen tubes. We hypothesize that some of the major pathways for unconventional protein secretion (including soluble cytosolic proteins) in pollen tubes is through multivesicular bodies and translocation into exosomes to reach the plasma membrane, apoplast, and the extracellular matrix.

### Unconventionally secreted pollen tube protein TCTP remarkably finds entry into the ER and Golgi and co-localizes with the Ole1 exosome marker

The multiple pathways for unconventional protein secretion have largely remained undefined. To demonstrate unconventional protein secretion and define the pathway of secretion, we chose a leaderless secreted protein, translationally controlled tumor protein (NtTCTP), based on its lack of a predicted N-terminal SP, its ambiguous localization prediction, the prediction that it is secreted by secretomeP (neural network (NN) score = 0.6), its absence of transmembrane helices, and the prediction that it resides “outside” with highly hydrophobic residues (Additional file 13: Figure S8). NtTCTP is an 18.7 kDa protein containing a pfam TCTP domain that is similar to *Arabidopsis* TCTP/TCTP2 (Additional file 13: Figure S8). Using an in silico approach, TCTP was predicted to localize in the cytosol and be associated with the GO terms multivesicular bodies and extracellular space. We expressed NtTCTP-GFP under the CaMV 35S promoter and observed that NtTCTP localizes to the apoplast as well as the cytosol and the nucleoplasm of tobacco leaf epidermal cells (Fig. 4p–s). We verified the apoplastic localization by plasmolysis and co-expression with viral AVR2-RFP apoplastic protein (Fig. 4p–s). Next, we co-expressed NtTCTP with ER and ER–TGN markers. Remarkably, we observed that NtTCTP partially co-localized with the ER retention marker HDEL-mCherry in the ER lumen and ER lamina (Fig. 5c, c-i, d). Subsequently, NtTCTP co-expression with the soybean Golgi vesicle marker GmMan1-mCherry showed over 89 % co-localization (*n* = 296 confocal slices), suggesting that NtTCTP is translocated from the ER to Golgi vesicles (Fig. 5e, ei). These unexpected observations imply the existence of a non-canonical motif within the NtTCTP sequence (or the presence of an ER TCTP chaperon protein) that enables TCTP to enter the ER and progress through the Golgi and TGN secretory pathway.

We then assessed how NtTCTP could reach the apoplast. In mammals, TCTP accumulation is regulated at the transcriptional and translational levels through reciprocal repression with p53 [49]. In humans, TSAP6 facilitates the unconventional secretion of TCTP outside the cell to participate in inflammatory responses [50]. Its secretion was not affected by brefeldin A (BFA) or monesin treatment and it localized in secreted nanovesicle exosomes [50]. We have used the Oleisin 1 (Ole1) protein from *Arabidopsis* as an exosome marker, a homolog of which was detected in secreted exosomes of olive pollen tubes [51]. Co-expression of NtTCTP-GFP with Ole1-mRFP in *N. benthamiana* leaf epidermal cells revealed a remarkable average overlap of > 75 % between NtTCTP-GFP-labeled granules and Ole1-mRFP-labeled granules (Fig. 5g, h). The identified exosome-like aggregates showed diverse morphological features, suggesting several levels of likely aggregation (Fig. 5g). Although independent verification of the NtTCTP association with exosomes will be necessary, our initial results nevertheless imply that NtTCTP could reach the apoplast via secreted nanovesicle exosomes. Remarkably, when we compared the proteome of secreted exosomes from olive pollen tubes [51] with our tobacco SIV secretome, we observed a significant 68.8 % (35/51) overlap, of which 94.3 % were unconventionally secreted proteins (Additional file 18: Table S7). It will be critical to confirm whether NtTCTP secretion to the apoplast is indeed mediated by nanovesicles and whether its infiltration into the conventional secretion pathway is a phenomenon shared with other unconventionally secreted proteins.

### Secretion of NtPsCRP2 is partially compromised in TGN *yip4a-1;4b* double mutants

**Fig. 6:**
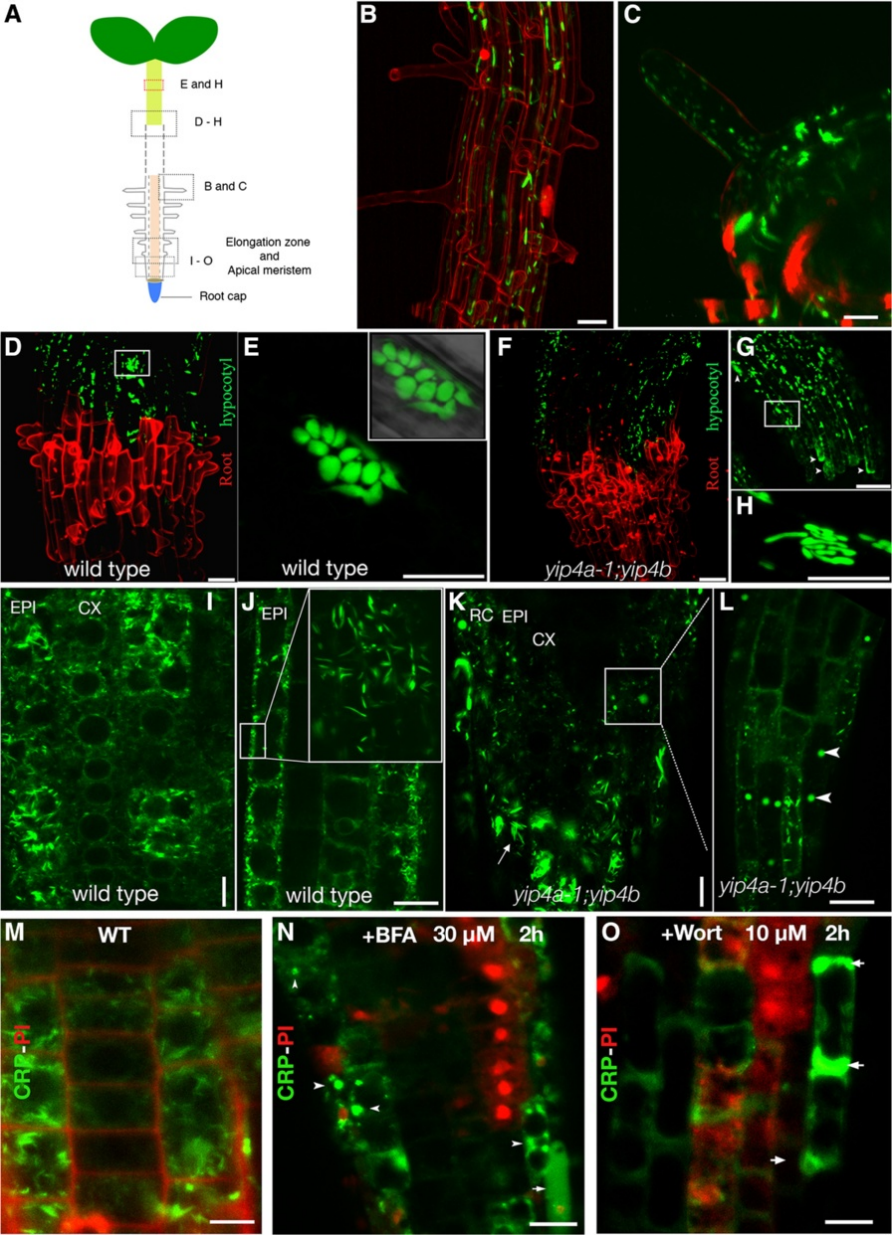
Simultaneous knockdown of YIP4A/B perturbed NtPsCRP2 secretion in seedlings. **a** An *Arabidopsis* seedling showing regions analyzed in this study. **b, c** NtPsCRP2-GFP localized less frequently in young root hairs but more frequently in mature root hairs, both in the wild type and in the *yip4a-1;yip4b* double mutant. **d** NtPsCRP2 secretion in 4-day-old etiolated wild-type seedlings showing localization in epidermal cells of the hypocotyl. The secretion was restricted at the hypocotyl–root junction. Propidium iodide (*red*) marks the beginning of the root. **e** Close-up of NtPsCRP2-GFP in elongated epidermal cells of the hypocotyl showing accumulation in spherical, aggregated endosomal-like vesicles. **f, g** Conversely, in *yip4a-1;yip4b* seedlings, NtPsCRP2 secretion was severely distorted, showing diffuse localization and non-uniform protein aggregates (*arrowheads*). **h** The spherical marked endosomal aggregates observed in the wild type were substituted with rod-like labeled organelles in *yip4a-1;yip4b* hypocotyl. **i** NtPsCRP2 localization at the root tip of wild-type seedlings marking secretory organelles. *EPI* epidermis, *CX* cortex. **j** Magnified root epidermal cells from wild type showing NtPsCRP2-marked organelles constrained to the vicinity of the plasma membrane (*white rectangle*) and also localized in ER-body-like organelles (*inset*). **k, l** In *yip4a-1;yip4b*, NtPsCRP2-GFP localization in secretory organelles at the root apical meristem appeared largely disorganized, showing severe organelle aggregations resembling BFA-like compartments (*arrowheads*). The ER-body-like localization (*arrow*) was maintained, suggesting no effect on ER-body biogenesis in *yip4a-1;yip4b* mutants. **m–o** Perturbed secretion of NtPsCRP2-GFP was also recapitulated following BFA or wortmannin treatment. *Arrowheads* show NtPsCRP2-GFP BFA compartments, *arrows* point to diffusely aggregated NtPsCRP2-GFP aberrant secretion. *RC* lateral root cap *PI* propidium iodide stain, *WT* wild type. Scale bars = 10 μM

To provide genetic evidence of the pathway for NtPsCRP2 secretion as well as establish whether conventionally secreted pollen tube proteins follow a conserved pathway of secretion in the sporophyte, we ectopically expressed NtPsCRP2-GFP in *Arabidopsis yip4a-1;4b* double mutant plants. YIP4A and YIP4B are conserved YPT/RAB GTPase-interacting proteins that form a complex with ECHIDNA (ECH) at the TGN and participate in the recycling of RAB-GDP during retrograde vesicle assembly and plasma membrane vesicle tethering [52]. Knockdown of both isoforms perturbs ER–TGN protein secretion and causes severe defects in plant development [52]. At the root-hair maturation zone, NtPsCRP2-GFP localized in mature root hairs and rarely in root hair initials, both in wild type and in *yip4a-1;yip4b* seedlings (Fig. 6a–c). In hypocotyl epidermal cells of 4-day-old etiolated wild-type seedlings, secreted NtPsCRP2-GFP formed uniform spherically shaped aggregates likely derived from endomembrane compartments that were restricted at the stem–root junction (Fig. 6d, e). Conversely, in *yip4a-1;yip4b* double mutants, NtPsCRP2-GFP was localized in deformed, rod-shaped endomembrane compartments instead of the spherical endomembrane aggregates observed in the wild type (Fig. 6f–h). Further, at the stem–root junction, NtPsCRP2-GFP showed diffuse localization and non-uniform protein aggregates (Fig. 6g, arrowheads) that were not observed in wild-type seedlings. In roots, NtPsCRP2-GFP localized predominantly in the epidermal cell layer in uniform endomembrane/secretory vesicles at the root apex, root elongation zone, and root hair zone (Fig. 6i, j). Interestingly, in the epidermal cell layer at the root apical zone, NtPsCRP2-GFP localized in spindle-shaped ER-body-like structures (Fig. 6j, inset). Live cell imaging of epidermal cells at the root elongation zone at 16.7 and 2.7 seconds per frame revealed that NtPsCRP2-GFP-labeled vesicles displayed three main types of dynamic behaviour: (1) quiescent or permanently membrane-tethered vesicles; (2) membrane-tethered vesicles that re-initiated mobility; and (3) mobile vesicles followed by membrane tethering (Additional file 14: Figure S9). In *yip4a-1;yip4b* roots, NtPsCRP2-GFP localized in endomembrane aggregates that were not uniformly distributed and formed larger aggregates in epidermal cells that resembled BFA compartments (Fig. 6k, l). However, the NtPsCRP2 localization in spindle ER-body-like structures (Fig. 6k, arrow) was maintained. We independently observed similar secretion defects of NtPsCRP2-GFP in roots following 2-h treatment of 4–5-day-old seedlings with BFA or wortmannin drugs (Fig. 6m–o). Our observations emphasize that NtPsCRP2-GFP is secreted through the ER–TGN pathway in vegetative tissues and that the recycling of NtPsCRP2 secretion to the proximity of the plasma membrane and endocytic secretory vesicles is perturbed in *yip4a-1,4b* double mutant plants. Since NtPsCRP2 posses an N-terminal SP and is secreted by the pollen tubes, this suggests that the conventional pathway is conserved between the gametophytic and sporophytic tissues. Further, it is likely that NtPsCRP2 is maintained in the secretory pathway until the appropriate signal (likely derived from the female reproductive tissues) is perceived for its secretion to the extracellular matrix.

### Secretion of LORELEI-like GPI-anchored protein 3 (LLG3) is perturbed in *yip4a-1;yip4b* pollen tubes

**Fig. 7:**
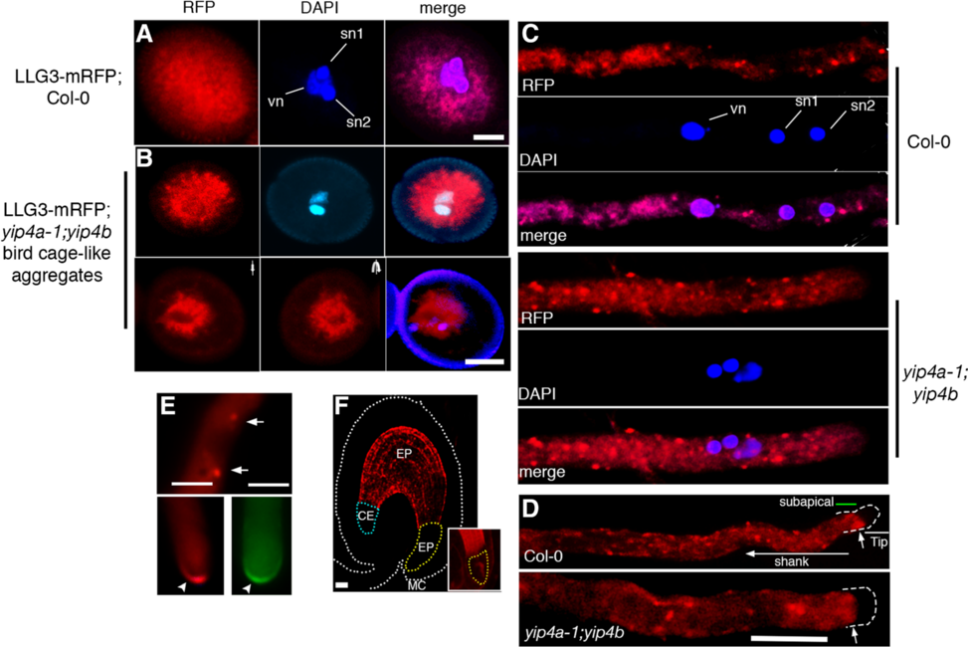
LLG3 secretion is compromised in *yip4a-1;yip4b* mutant pollen and pollen tubes. **a** Subcellular localization of LLG3-mRFP under native promoter showing cytosolic foci-like aggregates. *vn* vegetative cell nucleus, *sn* sperm cell nuclei. **b** *Top*: in the *yip4a-1;yip4b* mutant, the majority of the pollen grains displayed distinct cytosolic aggregates different from those observed in the wild type. *Bottom*: z-stack projection of 25 confocal slices showing distinct vegetative cell “bird cage-like” localization that was not observed in wild-type pollen grains. **c** Localization of LLG3-mRFP in wild-type pollen tubes grown in vitro for 16 h showing localization in likely secretory vesicles of uniform size and partially in the pollen tube ER (*top three panels*). In contrast, *yip4a-1;yip4b* mutant pollen tubes displayed a significantly higher frequency of LLG3-mRFP-marked endomembrane aggregated vesicles of variable sizes (*bottom three panels*). However, the ER localization was not greatly affected. **d** LLG3-mRFP also specifically accumulated at the subapical domain (*top panel*, *arrow*). This accumulation was also not significantly affected in *yip4a-1;yip4b* mutant pollen tubes (*bottom panel*). **e** In tobacco pollen tubes, similar localization in secretory vesicles was also observed (*top panel*, *arrows*) and occasionally LLG3-mRFP as well as LLG3-sGFP showed pollen tube tip-specific localization (*bottom panels*, *arrowheads*). **f** Absence of LLG3-mRFP protein in *Arabidopsis* unfertilized ovules and embryos soon after fertilization (18 h after pollination). Rarely, LLG3-mRFP could be detected at the embryo proper (*EP*) zone in some fertilized ovules. *MC* micropylar, *EP* endosperm proper, *CE* chalaza end endosperm. Scale bars for **a** and **b** = 30 μM and for **c–e** = 10 μM

To provide evidence for pollen tube protein secretion among the identified candidate secreted proteins, we analyzed the subcellular localization of GPI-anchor protein LLG3 and its likely secretion pathway. We fused a genomic fragment (AT4G28280) of the *Arabidopsis* ortholog of *N. tabacum* LLG3, including its putative promoter, with red fluorescent protein (RFP) inserted 12 amino acids upstream of the predicted GPI-anchor site. Microarray analysis [53] identified *Arabidopsis* LLG3 to be predominantly expressed in pollen and pollen tubes with elevated expression in pistils 8 h after pollination (Additional file 19: Figure S10). Semi-quantitative RT-PCR results showed tobacco LLG3 to be expressed in SIV pollen tubes as well as in unfertilized ovules of *N. tabacum* (Fig. 1g). It is not clear if this differential expression of LLG3 between the two species is significant. Expression of chimeric proLLG3-LLG3:RFP-GPI anchor in *Arabidopsis* showed localization in distinct compartments in the cytosol of mature *Arabidopsis* pollen grains partially resembling the ER network (Fig. 7a). In *yip4a-1;4b* mature pollen grains, LLG3-RFP distribution showed a distinct vegetative cell “bird cage-like” polarised localization, suggesting perturbed secretion or localization of LLG3 (Fig. 7b). After 12 h of in vitro wild type pollen tube growth, LLG3-RFP-GPI localized in secretory vesicles, in the vegetative cell cytosol, and partially in the likely ER network (Fig. 7c). In contrast, in *yip4a-1;4b* mutant pollen tubes, LLG3-RFP-GPI distinctively localized in non-uniform, aggregated endomembrane-derived vesicles of variable sizes (Fig. 7c). However, the ER localization was not affected in the mutant background (Fig. 7c). Further, LLG3-RFP-GPI accumulated at the sub-apical domain, both in wild-type and in *yip4a-1;4b* mutant pollen tubes, but we could not observe localization at the pollen tube tip and the plasma membrane (Fig. 7d). In tobacco pollen tubes, LLG3-RFP-GPI as well as LLG3-GFP-GPI occasionally showed exclusive tip localization as well as granular formation in likely secretory vesicles (Fig. 7e). In the *Arabidopsis* female gametophyte, we could not detect LLG3-RFP-GPI expression in unfertilized ovules, although we rarely observed weak signal at the embryo-proper region approximately 18 h after pollination. Our results verify secretion by the pollen tube of candidate pollen tube-secreted proteins identified in this study and provide evidence for the requirement for YIP4A/4B isoforms for proper LLG3 secretion through the ER–TGN pathway during pollen tube growth.

### The ER–TGN secretory mutant *echidna* is severely infertile, partly attributable to a lack ovule-targeting competence in the pollen tube

**Fig. 8:**
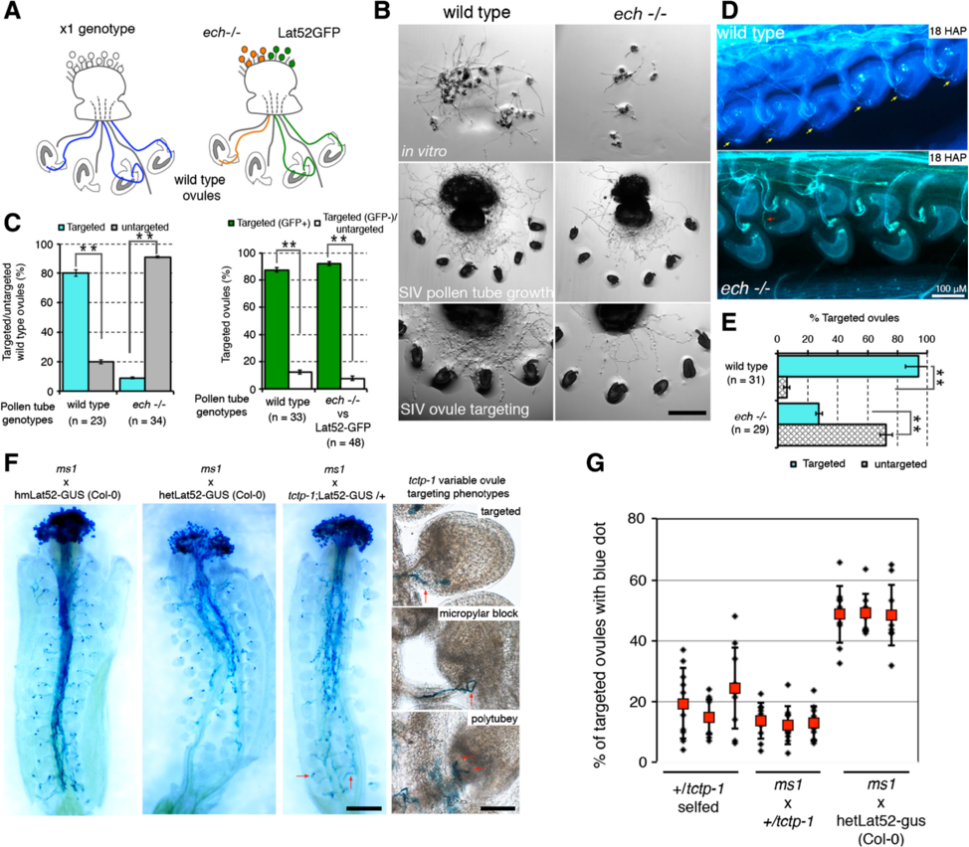
TCTP- and ECHIDNA-deficient pollen tubes consistently fail to target ovules. **a** The SIV ovule-targeting assay. Pollen tube targeting competence was assessed using a single pollen genotype or in a one-to-one genotype competition assay. **b** *ech*-/- pollen grains showed reduced but adequate pollen tube germination in vitro as well as semi*-*in vivo but showed significantly reduced ovule-targeting competence semi*-*in vivo. **c** Quantification of pollen tube ovule-targeting competence semi-in vivo of *ech*-/- plants in a single genotype or mixed genotype competition assay. *n* is the number of pistil explants assessed, *asterisks* denote significant differences assessed with Student’s *t*-test (*p* < 0.01). **d** Aniline blue stain of wild-type and *ech*-/- pollinated wild-type pistils 18 h after pollination (*HAP*) showing near complete lack of ovule targeting in *ech*-/- pollinated pistils (*red arrow*) compared with wild type-pollinated pistils (*yellow arrows*), verifying the semi-in vivo observations in **c. e** Frequency of ovule targeting by pollen tubes in aniline blue-stained pistils. All score variances (*asterisk*) are statistically significant (Student’s *t*-test, *p* < 0.01). Error bars represent standard deviation (stdv). **f** Blue dot assay by GUS-staining of *ms1* pistils pollinated with wild-type pollen grains homozygous or heterozygous for Lat52-GUS and Lat52-GUS;*tctp-1/+* 18 HAP. *Red arrows* point to *tctp-1* mutant pollen tubes targeting ovules at the bottom of the pistil, suggesting *tctp-1* pollen tube growth is not greatly impaired. *Insets*: variable mistargeting phenotypes observed in *tctp-1*/+ pollinated pistils. **g** Counts of “blue dots” revealed ovule targeting was greatly impaired in *tctp*-deficient pollen tubes

Since 15.95 % of the pollen tube secretome comprises proteins secreted through a conventional ER–TGN pathway, we investigated whether loss of ECHIDNA function, an interaction partner of YIP4A/4B [54], would also compromise pollen tube–ovule crosstalk and ovule-targeting competence. Earlier observations revealed that *ech-*/- plants are male semi-sterile, producing only a handful of pollen grains with reduced viability and ability to grow pollen tubes [55]. Despite this, the study showed that >42 % of the tetrads from *ech*-/- plants had three to four viable pollen grains. Moreover, *echidna* mutation can be propagated as homozygous, indicating that some *echidna* pollen grains can germinate a pollen tube and undergo fertilization with *echidna* mutant ovules. Here, we have also shown that *ech*-/- pollen grains can germinate pollen tubes in vitro as well as semi*-*in vivo, although at a reduced growth rate compared with wild-type pollen tubes (Fig. 8a, b). We have independently shown that pollination of wild-type pistils by *ech*-/- pollen grains produced adequate amounts of viable seeds that are able to germinate into plantlets; these amounts were greater than from *ech*-/- selfing plants, suggesting that the *echidna* mutant has reduced fertility in both gametophytes. These results provide evidence that *echidna* mutation can be transmitted through the male (albeit at very low frequency), supporting the ability of *ech*-/- pollen grains to grow a pollen tube and occasionally target ovules for successful fertilization.

**Fig. 9:**
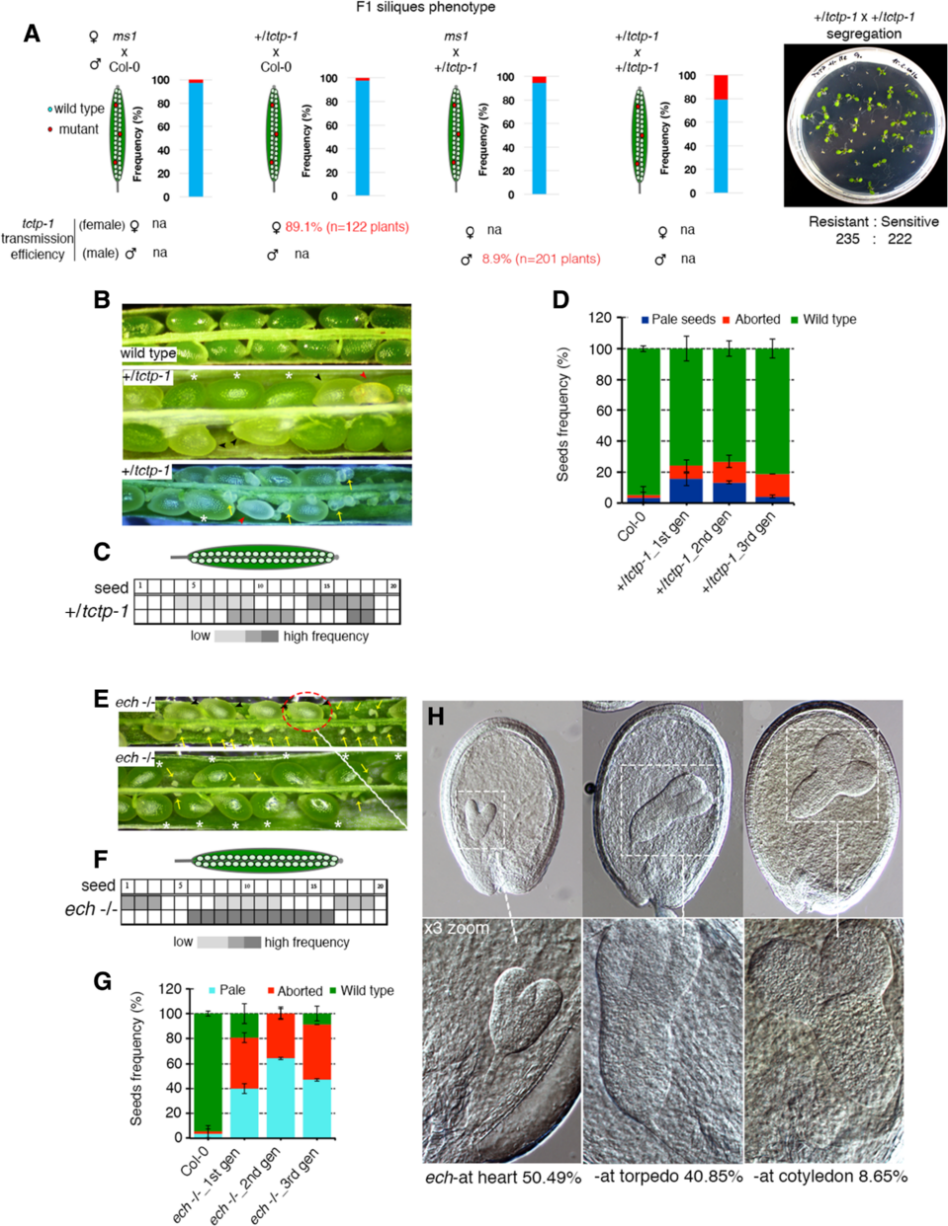
Loss of TCTP and ECHIDNA functions also impairs post-fertilization events. **a** Reciprocal test crosses of the *tctp-1* mutation showing low penetrance male-specific phenotypic induction in F1 siliques as well as significantly reduced male transmission efficiency scored by PCR genotyping as well as GUS-staining of LAT52-GUS-tagged T-DNA insertion. The micrograph on the *far right* shows seedling segregation of self-fertilized +/​*tctp-1* plants, supporting the near complete block of *tctp-1* allele transmission through the male. **b** Dissected siliques of wild-type and self-fertilized +/​*tctp-1* plants. *Asterisks* indicate wild-type-like seeds, *black arrowheads* indicate underdeveloped chlorotic mutant seeds, *red arrowheads* and *yellow arrows* show arrested embryos with failed embryogenesis soon after fertilization as well as unfertilized ovules. **c** Frequency and positioning of mutant seeds within dissected siliques. The random distribution of mutant seeds within +/​*tctp-1* siliques suggests *tctp-1* mutant pollen tube growth is competent. **d** Frequency of *tctp-1* mutant seed phenotypic classes observed (n = 25 siliques per line). Error bars represent standard deviation (stdv). **e** Dissected siliques of self-fertilized *ech*-/- plants showing the high frequency of failed fertilization, defective embryogenesis, as well as “wild-type-like” seeds (*asterisks*). **f** Frequency and random positioning of *ech* mutant embryos/unfertilized ovules within *ech*-/- self-fertilized siliques implying *ech* pollen tubes can grow to the base of the pistil and target ovules for fertilization. **g** Frequency of mutant seed phenotypic classes observed (n = 25 siliques per line). Error bars represent standard deviation (stdv). **h** Embryogenesis lethality in ECHIDNA-deficient embryos showing stages and frequency of embryo arrest. Scale bars = 10 μM. *gn* germ cell nuclei, *na* not applicable, *sn* sperm cell nuclei, *vn* vegetative cell nuclei, *wt* wild type

To emphasize the fertility defects of *ech*-/- plants, we investigated the guidance and ovule-targeting ability of the viable *echidna* pollen tubes. We used *ech*-/- pollen grains to hand-pollinate pistils and score their ovule-targeting competence 18 h after pollination (HAP) (Fig. 8a–e). Critically, we used the SIV assay to score only pollen tubes that emerged from the cut pistils on their ability to target wild-type ovules (Fig. 8a, b). This way, we eliminated the non-viable population of *ech*-/- pollen grains and dealt only with the pollen grains that could germinate and grow pollen tubes through the cut pistil (Fig. 8b). Of the handful of viable *ech-*/- pollen grains produced and able to germinate, we noticed that they were vastly outcompeted by wild-type pollen tubes labeled with LAT52-GFP in targeting ovules for fertilization in a mixed genotype competition assay or were poorly targeting in self-pollinated *ech-/-* pistils (Fig. 8c–e). The *ech-/-* pollen tube ovule-targeting incompetence was further emphasized by the fertility defects and lack of ovule fertilization observed in dissected mature *ech-/-* siliques (Fig. 9e). On average, only 10 % of the ovules in *ech-*/- plants were fertilized and able to develop into “wild-type-like” seeds (Fig. 9e–g). Moreover, nearly 50 % of the self-fertilized *ech-/-* ovules showed embryo developmental defects, the majority of the embryos arresting at the heart and torpedo stages (Fig. 9h). The penetrance of this infertility was greatly variable between siliques as well as between individual plants, with some siliques producing almost no seeds (Fig. 9h). ECHIDNA protein is required for the secretion of secGFP but not BR11 or PIN2 auxin efflux carrier and the *ech-/-* mutant effect phenocopied concanamycin A defective secretion [54]. Earlier, we showed that the secretion of both the pollen tube proteins NtPsCRP2 and LLG3 was perturbed in *yip4a-1;4b* double mutants (Figs. 6 and 7). Since ECH forms a complex with YIP4A/B in the TGN, both *ech* and *yip4a/yip4b* double mutants could widely affect secretion of several pollen tube-secreted proteins essential for pollen tube growth, pollen tube–ovule crosstalk, fertilization, as well as embryogenesis in flowering plants, contributing to the infertility observed in *echidna* mutant plants.

### Secreted TCTP protein is required for pollen tube guidance and ovule targeting

Loss of function of TCTP was previously reported to impact on pollen tube growth [56] and plant fertility [57] in *Arabidopsis*. Here, we have incorporated our findings on NtTCTP secretion by the pollen tube following stylar penetration and independently investigated the phase of TCTP function during fertilization. We analyzed a T-DNA knockout line of the tobacco TCTP homolog (74 % identity, e-value 2.0E-85; Additional file 13: Figure S8) from *Arabidopsis* (AT3G16640, SAIL_28_C03, *tctp-1*) and investigated the ovule-targeting ability of its pollen tube and its seed production. Surprisingly, we did not observe the pollen tube growth defects reported by Berkowitz et al. [56] (Additional file 13: Figure S8). We exploited the GUS marker that co-segregates with the T-DNA insertion and performed a blue dot assay by GUS staining in pistils 18 HAP. We observed that *tctp-1* mutant pollen tubes from +/*tctp-1* plants poorly target ovules for fertilization (Fig. 8f). We noticed that *tctp-1* mutant pollen tubes can grow normally to the bottom of the pistil and occasionally target ovules, suggesting pollen tube growth in the *tctp-1* mutant is normal (Fig. 8f, arrow) Up to 35 % targeting variability was observed between pistils and between individual plants, suggesting variable *tctp-1* phenotypic penetrance (Fig. 8g). Transmission analysis confirmed that *tctp-1* mutation is poorly transmitted through the male (8.9 % transmission efficiency) but is normally transmitted through the female (Fig. 9a). Further, reciprocal pollination revealed that the mutant embryo lethal phenotype is exclusively induced by mutant pollen tubes at low penetrance (two weeks post-pollination) but not by the *tctp-1* mutant ovules (Fig. 9). Collectively, these results suggest that TCTP is essential for proper pollen tube guidance and ovule targeting as well as embryogenesis.

Dissected mature siliques from self-fertilized +/*tctp-1* plants revealed severe infertility and reduced seed set (Fig. 9b–d). The appearance of mutant seeds was randomized in siliques, suggesting equal competitiveness between the mutant and wild-type pollen tube growth (Fig. 9c). We observed an average of 12.3 % aborted ovules early post-fertilization and additionally 10.9 % of seeds were aberrantly chlorotic (Fig. 9d). In total, 44.5 % of the embryos with chlorotic seeds were arrested at the heart stage, with 37 % terminating at the torpedo stage and 18.5 % at the premature cotyledon stage (Fig. 9d). Our results suggest a critical role of TCTP in pollen tube guidance, ovule targeting, the events leading to fertilization, and the early stages of embryogenesis.

## Discussion

Navigation of the pollen tube through several female sporophytic tissues to reach the ovule for fertilization (pre-ovular guidance) relies on guidance mediated by proteins, peptides, and other chemoattractants secreted by the female reproductive tissues [58]. This molecular dialog has emerged as an important bottleneck for unfavorable fertilization and as a pre-zygotic barrier for interspecies hybridization. A compendium of recent studies have identified a handful of secreted peptides from female synergid cells of *T. fournieri*, *Z. mays*, and *Arabidopsis* as pollen tube attractants [3–5, 59]. Another recent study also reported that CENTRAL CELL GUIDANCE protein (CCG) together with its interaction partners, CCG BINDING PROTEIN 1 (CBP1), mediator complex (MED), and central cell-specific AGAMOUS-transcription factors, co-regulate a subset of cysteine-rich proteins (CRPs), including pollen tube attractant LURE1 [9]. Only two pollen tube receptor proteins have been identified so far as playing a role in pollen tube guidance, LIP1 and COBL10 [31, 60]. Such slow progress has been attributed to the lack of accessibility of the pollen tubes within the pistil to capture and identify proteins secreted by the pollen tubes during their interaction with pistil tissues. In vitro approaches are feasible but pollen tubes grown in vitro do not acquire the competence to respond to female guidance signals [16] and develop different pollen tube physiology [12, 15, 61–63]. In this study we have successfully demonstrated the application of a previously developed pollen tube semi-in vivo technique (SIV-PS) [14] combined with gel- and label-free quantitative LC-MS/MS as a powerful assay for the genome-wide characterization of pollen tube-secreted proteins following its penetration through stigma and style. The SIV-PS tool offers access to pistil-stimulated pollen tube-secreted proteins with minimal contamination. We show that by coupling SIV-PS with gel- and label-free LC-MS/MS together with a structured bioinformatics workflow, one could quantitatively identify known pollen tube-secreted proteins as well as establish novel proteins with previously unknown extracellular functions. Using this technique, we demonstrate that the SIV-PS is unique from that of in vitro germinated pollen tubes (36.3 % overlap), emphasizing the requirement of pistil factors in rendering pollen tubes competent to target the ovule.

We have demonstrated the efficacy of the SIV-PS-identified proteins through subcellular localization prediction and *in planta* demonstration and investigated the undertaken secretory pathways and the involvement of key regulators of secretory pathways and pollen tube-secreted proteins in plant fertility and seed setting (Figs. 8 and 9). Among the identified secreted proteins are supposed ligands and receptors of the pollen tube that could facilitate cell–cell communication with the female reproductive and gametophytic tissues during ovule targeting by the pollen tube and fertilization. These include the plant defensin subfamily, CRPs, LORELEI-like GPI-anchored 3 (LLG3), thionin-like protein, RNases, lipid transfer proteins (LTPs), pollen Ole-e-allergen, arabinogalactans, pectinases, and invertases. Correlation analysis between the pollen tube secretome and transcript abundance revealed the two processes are uncoupled, as shown by the lack of linearity (Fig. 3). These observations clearly indicate that secreted proteins are subjected to multiple regulatory mechanisms at the post-transcriptional level prior to their secretion to the extracellular matrix. This lack of correlation has been reported in secretome studies of various human cancer types that deploy secretome techniques in search of biomarkers and cancer therapeutic targets ([64] and references therein). Therefore, in silico prediction alone for protein secretion provides only a rough guideline; instead, direct quantitative proteomic analysis of secreted proteins is necessary to identify and evaluate true protein secretion.

Further, we have demonstrated that conventional pathways play an essential role in pollen tube protein secretion. This was underscored by the defective secretion of the pollen tube proteins NtPsCRP2 and LLG3 in *yip4a-1;yip4b* double mutants, lack of pollen tube guidance and ovule-targeting competence in an *echidna* loss-of-function mutant, and the broader expression of several conventional pathway regulatory genes in tobacco SIV pollen tubes (Additional file 10: Figure S5). A similar role was previously demonstrated for the POD1 protein involved in ER protein folding [65] as well as CHX21 and CHX23, which maintain ER potassium homeostasis [66], both exclusively affecting pollen tube guidance.

We have shown that, despite the significance of conventional protein secretion (Additional file 20: supplementary text), the bulk of pollen tube-secreted proteins utilize unconventional secretory pathways [48, 67]. We propose that one pathway for unconventional secretion of pollen tube proteins is via bioactive nanovesicle exosomes. In animals, several cell types secrete exosomes and other types of vesicles (ectosomes and shedding microvesicles) for intercellular communication with neighboring cells, affecting gene expression and overall cell physiology [68, 69]. Similarly, exosome secretion was recently reported in olive pollen tubes [51]. Of great interest, our comparison of the proteome from the secreted exosome of olive pollen tubes with the tobacco pollen tube secretome revealed a significant 68.6 % (35/51) overlap (Additional file 18: Table S7). Astonishingly, 94.3 % of these shared proteins are secreted unconventionally and only 5.7 % were signal peptide (SP)-containing proteins. These include proteins involved in cell wall expansion, stress/defense, membrane transport, metabolism, signaling, and protein synthesis and processing. In support, we have demonstrated that TCTP is secreted to the apoplast and strongly co-localized with the exosome marker Ole1 from *Arabidopsis* (Figs. 4p–s and 5g–h). Our results suggest that pollen tube-secreted exosomes could account for the secretion of the majority of unconventionally secreted pollen tube proteins to the extracellular matrix. In animals, Notch ligand Delta-like 4 is secreted via exosomes and was shown to impact on neighboring cells without the conventional cell–cell contact and potentially at a longer range [70]. We propose that pollen tube-secreted exosomes could facilitate a latent mechanism for pollen tube long-distance signaling with the female reproductive tissues, possibly including crosstalk with ovules. We have shown that secretion of NtTCTP seems to follow the ER–TGN route, as shown by co-localization with ER and Golgi markers (Fig. 5c–e). This phenomenon has so far not been reported for unconventionally secreted proteins [28]. Secretion of unconventional proteins is known to be insensitive to BFA treatments, suggesting a Golgi apparatus bypass [71]. Perhaps it is questionable to base the absolute localization of NtTCTP in Golgi on co-localization with the Golgi marker GmMan1 alone. In *Arabidopsis*, the secretion of the unconventionally secreted protein hygromycin phosphotransferase (HYG^R^) is BFA-insensitive; however, it still requires the participation of a Golgi-localized synaptotagmin homolog, SYT2 [72]. Moreover, SYT2’s role in HYG^R^ unconventional secretion has been compared with the Golgi-localized GRASP function in the unconventional secretion of AcbA in *Dictyostelium* and alpha-integrin in *Drosophila* [71]. Therefore, the co-localization of NtTCTP with GmMan1 could suggest either that NtTCTP is not strictly localized in the Golgi apparatus or that GmMan1 is instead involved in the unconventional secretion of NtTCTP. Alternatively, NtTCTP could be truly localized in the Golgi apparatus and its unconventional secretion does not bypass the Golgi as in other examples of unconventionally secreted plant proteins. Future experiments will confirm precisely which route NtTCTP follows for its final phase of secretion to the apoplast.

A parallel pathway that could also facilitate unconventional protein secretion, particularly of soluble cytosolic proteins, is via secretory lysosomes [73]. Cofactors such as plant orthologs of animal caspases could facilitate secretion through lysosome membrane fusion and microvesicle shedding [73]. Another exciting proposal for an unconventional protein secretion pathway in plants is via a recently discovered exocyst positive organelle, EXPO [74]. Although EXPO co-localized with Exo70E2, a marker of the exocyst complex, its role in conventional protein secretion was ruled out based on the fact that EXPOs are not influenced by BFA or wortmannin and are unable to uptake the endocytotic marker FM4-64 [28]. EXPOs role in mediating unconventional protein secretion was demonstrated in the secretion of SAMS2 (S-adenosyl methionine synthetase 2) from the cytosol to punctate organelles that co-localized with Exo70E2 in the protoplast [74]. Further, EXPOs have been identified as double membrane organelles and fuse with plasma membrane to expel a single membrane vesicle to the apoplast [74]. It will be of great interest to establish whether EXPOs are also involved in unconventional secretion of pollen tube proteins alongside exosomes during male–female communication.

The bioinformatics analysis of the SIV-PS dataset also revealed unexpected phenomena. Proteins predicted to be palmitoylated at cysteine residues showed an extensive overlap with proteins predicted to undergo GPI-anchoring modification. This might be explained by the fact that cysteine palmitoylation of putative GPI-anchored proteins (GAPs) could facilitate increased affinity of GAPs for the plasma membrane prior to their secretion. Indeed, both COBRA-like 10, a pollen tube receptor protein, and Lorelei-like GPI-anchored 3 (LLG3, this study) were predicted to be palmitoylated at amino acids Cys206, 336, 337, and 433 for COBRA-like 10 and amino acids Cys7, 74, and 75 for LLG3. Furthermore, in animals, cysteine palmitoylation was shown to regulate raft affinity for the majority of the raft integral proteins, including GAPs [75, 76]. Because of the low proportion (14 %) and very low abundance of GAPs identified in this study, it will be important to perform GPI-protein enrichment of the pollen tube fractions to identify other potential pollen tube GPI-anchored receptors.

Using gel- and label-free quantitative LC-MS/MS at the genome-wide scale, our study has revealed for the first time the pollen tube proteins secreted in response to pollen tube interaction with the female reproductive tissues. These secreted proteins represent potential ligands and receptors for cell–cell signaling during pollen tube–pistil interaction. Most significantly, we speculate that pollen tubes might deploy exosome nanovesicles as a mechanism to facilitate long distance cell–cell communication with the female reproductive tissues. We have shown the utilization of ER–TGN pathways by pollen tube-secreted proteins in both the sporophyte and male gametophyte and demonstrated that perturbation of this pathway is detrimental for their secretion. Using the unconventionally secreted pollen tube protein NtTCTP, we have demonstrated that pollen tube secreted proteins are indispensable for pollen tube guidance and ovule targeting as well as for embryogenesis. We have emphasized the significance of pollen tube protein secretion activities by showing defective pollen tube guidance and ovule targeting ability and near-complete sterility following the knockdown of ECHIDNA. Identified secreted proteins from this study offer a direct demonstration of species-specific candidate ligand–receptor interaction partners with an emphasis on cell–cell signaling mechanisms during successful plant reproduction.

## Conclusions

We have identified 801 genome-wide pollen tube secreted proteins following pollen tube penetration through female reproductive tissues. Bioinformatics analysis revealed that the vast majority of the pollen tube secretome comprises unconventionally secreted proteins, predominantly small proteins of < 20 kDa. They are predominantly expressed in the male gametophyte and include glycoside-hydrolase, copper binding, RNA-binding, and proteolysis among the most frequent associated activities. Tagging of selected candidate proteins with fluorophores verified localization in secretory pathways and secretion to the extracellular matrix. Further functional analysis revealed pollen tube-secreted proteins as well as regulators of ER–TGN protein translocation as crucial players for proper pollen tube protein secretion, guidance of pollen tubes to the ovule, and embryogenesis. Our study has provided new insights into alternative pathways for unconventional protein secretion involving nanovesicle exosomes and for the first time provided access to newly identified pollen tube secreted proteins that could perceive guidance signals from the female reproductive tissues during cell–cell communication and plant sexual reproduction.

## Methods

### Preparation of tobacco pollen tube secretome using the SIV-PS approach

To capture pollen tube-secreted proteins following penetration through the pistil, we developed the following procedure. On day 1, flowers were emasculated one day before anthesis and netted for 24 h. On day 2, pollen grains stored at −20 °C were acclimatized to room temperature for 5 min and used for limited pollination of half of the emasculated flowers. On day 3, both pollinated and unpollinated (control) pistils were collected and excised at approximately 22 mm from the stigma shoulders (Additional file 1: Figure S1). Pistils were arranged in a “germination cup” (5 × 4 cm radius × height) filled with SMM-MES pollen germination media (0.175 M sucrose, 1.6 mM H_3_BO_3_, 3 M Ca(NO_3_)_2_.4H_2_O, 0.8 mM MgSO_4_.7H_2_O, 1 M KNO_3_, 23 mM MES, pH 5.9). Pollen tube germination was performed at 28 °C in a 70 % humid chamber for 24 h (Additional file 1: Figure S1). Proteins secreted into media were concentrated using a Millipore filter (Amicon, USA Ultra-2 Pre-Launch 10 K and 3 K), and final samples were stored at −80 °C. Final protein concentrations were measured by 2D-Quant kit (GE Healthcare, USA). Pistils with protruding pollen tubes were used for viability tests and microscopic analysis and the remaining pollen tubes were excised for RNA extraction. For a stepwise description, see [14]. A list of primers used for semi quantitative RT-PCR analysis is provided in Additional file 21: Table S8.

### FASP processing

Biological replicates of concentrated SIV-PS media (including negative controls from unpollinated pistils) were subjected to filter-aided sample preparation (FASP) [77, 78]. Samples containing about 5 μg of total protein were mixed with UA buffer (8 M urea in 100 mM Tris-HCl, pH 8.5), loaded onto the Vivacon 500 device with MWCO 10 kDa (Sartorius Stedim Biotech, Germany), and centrifuged at 14,000× g for 30 min at 20 °C. Before sample application, 5 μl of 1 % (w*/*v) polyethylene glycol 20,000 (PEG) was added onto the membrane. The retained proteins were washed with 400 μl UA buffer. The final protein concentrates kept in the Vivacon 500 device were mixed with 100 μl of UA buffer containing 50 mM dithiothreitol and incubated for 30 min at room temperature. After additional centrifugation, the samples were mixed with 100 μl of UA buffer containing 50 mM iodoacetamide and incubated in the dark for 30 min. After the next centrifugation step, the samples were washed three times with 400 μl UA buffer and three times with 200 μl of 50 mM NaHCO_3_. Trypsin (sequencing grade, Promega, USA) was added onto the filter and the mixture (total volume about 50 μl, PEG concentration about 0.1 % (w/v)) was incubated for 14 h at 37 °C. The tryptic peptides were finally eluted by centrifugation followed by two additional elutions with 50 μl of 50 mM NaHCO_3_. The final eluate was concentrated using a SpeedVac concentrator (Thermo Fisher Scientific) down to about 20 μl and transferred into a LC-MS vial containing 2.5 μl of 0.01 % PEG, with additional acidic “extraction” of peptides from FASP eluate test tube walls by 50 % acetonitrile (ACN) containing 2.5 % formic acid (v/v) and by 100 μl of 100 % ACN (each extraction step was done twice with 50 and 100 μl of the solution, respectively). The final solution was concentrated in a SpeedVac concentrator to < 25 μl and refilled to 25 μl with water.

### LC-MS/MS analysis of peptides from FASP

LC-MS/MS analyses of the peptide mixture were done using a RSLCnano system connected to an Orbitrap Elite hybrid spectrometer (Thermo Fisher Scientific, Waltham, MA, USA). Prior to LC separation, tryptic digests were online concentrated and desalted using a trapping column (100 μm × 30 mm) filled with 3.5 μm X-Bridge BEH 130 C18 sorbent (Waters, Milford, MA, USA). After washing the trapping column with 0.1 % formic acid , the peptides were eluted (flow 300 nl/min) from the trapping column onto an Acclaim Pepmap100 C18 column (2 μm particles, 75 μm × 250 mm; Thermo Fisher Scientific, Waltham, MA, USA) using the following gradient program (mobile phase A, 0.1 % FA in water; mobile phase B, ACN:methanol:2,2,2-trifluoroethanol (6:3:1; v/v/v) containing 0.1 % FA). The gradient elution started at 1 % of mobile phase B and increased from 1 to 56 % during the first 100 min (1 % in the first, 14 % in the 30th, 30 % in the 60th, and 56 % in 100th min), then increased linearly to 80 % of mobile phase B in the next 5 min, remaining at this state for the next 15 min. Equilibration of the trapping column and the column was done prior to sample injection into the sample loop. The analytical column outlet was directly connected to the Nanospray Flex Ion Source (Thermo Fisher Scientific, Waltham, MA, USA).

MS data were acquired in a data-dependent strategy selecting up to the top 20 precursors based on precursor abundance in the survey scan (350 − 1700 m/z). The resolution of the survey scan was 120,000 (400 m/z) with a target value of 1 × 10^6^ ions, one microscan and maximum injection time of 200 ms. Low resolution CID MS/MS spectra were acquired with a target value of 10,000 in rapid CID scan mode with the m/z range adjusted according to actual precursor mass and charge. MS/MS acquisition in the linear ion trap was carried out in parallel to the survey scan in the Orbitrap analyzer by using the preview mode. The maximum injection time for MS/MS was 150 ms. Dynamic exclusion was enabled for 45 s after one MS/MS spectra acquisition and early expiration was disabled. The isolation window for MS/MS fragmentation was set to 2 m/z.

Two LC-MS/MS analyses in total were done for each sample (using 10 out of the 25 μl of the final solution). The second LC-MS/MS analysis was done with exclusion of m/z masses already assigned to peptides from the target database (FDR < 1 %) based on the first LC-MS/MS analysis. Mass tolerance for m/z exclusion was set to 10 ppm and the retention time window to 2 min. The two resulting raw files for each sample were searched as a single data set to obtain complete identification results for each sample.

The analysis of the mass spectrometric RAW data files was carried out using the Proteome Discoverer software (Thermo Fisher Scientific; version 1.4) with in-house Mascot (Matrixscience, London, UK; version 2.4.1) and Sequest search engines. Mascot MS/MS ion searches were done against the concatenated UniRef100 protein database for Solanaceae, Brassicaceae, and Fabaceae taxonomies (downloaded on 2014-02-26 from http://www.uniprot.org/) and an in-house tobacco protein database based on sequencing data (total number of protein sequences 624,436). A modified cRAP database (downloaded from  http://www.thegpm.org/crap/) was searched in parallel for detection of contaminants. Mass tolerance for peptides and MS/MS fragments was 5 ppm and 0.5 Da, respectively. Oxidation of methionine and deamidation (N, Q) as an optional modification, carbamidomethylation of C as a fixed modification, and two enzyme miscleavages were set for all searches. Percolator was used for post-processing of database search results. Peptides with a FDR (*q*-value) <1 %, rank 1, and with at least six amino acids were considered. Label-free quantification using protein group area calculation in Proteome Discoverer was used (Top3 protein quantification) [17, 18]. Parts per million (ppm) values for all or potentially secreted proteins were calculated as protein group area divided by the sum of areas of all or potentially secreted protein groups (molar ratio; expected to be directly proportional to protein amount in the original samples) multiplied by 10^6^, respectively.

Following label-free quantitative LC-MS/MS, protein groups were considered secreted based on Top3 protein algorithms stipulating that (1) the ratio of the calculated median of a protein group in a sample to the average median of the two control unpollinated samples was > 3 (cell “CP1” of Additional file 3: Table S1) and (2) the number of peptides was, at a minimum, 3 and that the protein group must be up-regulated in at least two SIV-PS samples (column “CS, U > 2). For comparison, individual protein accessions from replicates of all sample types were combined into supergroups (SGs) and reported in SG column Additional file 7: Table S4. Proteins were put into the same SG if they were reported in sample type replicate report as alternative proteins. For more information see actual reports and comments within tabs. To compare in vitro secreted proteins and the respective in vitro total proteomes, only protein groups with a minimum of three peptides and present in at least one replicate were used. All accessions from the respective groups that fulfill the above criteria are provided in Additional file 7: Table S4 with separate group columns for each pairwise comparison and accessions that are unique or intersect with the paired sample.

### Alcohol dehydrogenase enzyme assay

Endogenous ADH activity was measured in unpollinated controls, SIV-PS samples and in SIV pollen tubes total extract (SIV-PP) to estimate the extent of cytosolic protein contamination. As a control, the total soluble protein fraction from the respective pollen tubes was extracted using RIPA extraction buffer (Tris 50 mM, pH 8.0, 150 mM NaCl, 0.1 % SDS, 0.5 % sodium deoxycholate, Triton X-100, 1 mM PMSF) and protein concentrations were quantified using a 2D-Quant kit (GE Healthcare, USA). The reaction mixture for the ADH assay was composed of 50 mM Tris, pH 9.0, 0.867 mM NADþ, 20 % ethanol (Sigma, USA), and equal aliquots of protein extracts from each samples. The assay was repeated with three biological replicates. The increase in absorbance at 450 nm was monitored every 5 min for 45 min. The ADH activity was expressed as nmole/min/ml (milliunits/ml) for 1.0 μmole NAD^+^ to NADH reduction per minute at pH 8.0 and 37 °C, per aliquot of protein.

### Immunoblot protein detection

Total proteins from transiently transformed *N. benthamiana* leaves 48 h post-transfection or those from SIV and in vitro tobacco pollen tubes were extracted using RIPA extraction buffer (50 mM Tris pH7.4, 150 mM NaCl, 0.5 % Na-deoxycholate, 1.0 % Triton X-100). Protein samples were quantified using a 2D-Quant kit (GE Healthcare, USA), resolved by one-dimensional SDS-PAGE and blotted using the semi dry technique (e-blot, GeneScript) onto nitrocellulose membranes (GE Healthcare, USA). After 30 min blocking, blots were probed with a 1:1000 dilution of rabbit monoclonal anti-GFP antibody raised against sGFP (ChromoTek, Germany) overnight at 4 °C followed by a 1:30,000 dilution of rat anti-rabbit IgG conjugated to alkaline phosphatase (Sigma USA). Chemiluminescence was developed using BCIP (5-bromo-4-chloro-3-indolylphosphate, final concentration 165 ng/ml) and NBT (nitro blue tetrazolium chloride, final concentration 33 ng/ml) developing solutions in AP buffer (100 mM Tris-Cl pH 9.5, 100 mM NaCl, 5 mM MgCl_2_). Blot images were captured and analyzed using a Syngene G:Box EF imaging system (Syngene, UK).

### Concavalin A-coupled horseradish-peroxidase N-glycosylation test

Total proteins (40 μg per sample) were resolved by one-dimensional SDS-PAGE in quadruple replicates. Proteins were transferred onto nitrocellulose membrane and stained with Ponceau Red stain (Sigma, USA) for visualizing protein loading control. Membranes were de-stained, washed in TTBS buffer (500 mM NaCl, 80 mM Tris.HCl, pH 7.6, 0.1 % Tween 20) for 1 h and incubated in TTBS buffer supplemented with 25 μg/ml concavalin A for 1 h. Membranes were then washed for 30 min in TTBS and incubated for 30 min in TTBS with 50 μg/ml horseradish peroxidase before being washed again in TTBS for 45 min. N-glycosylated protein bands were visualized with detection buffer (45 mg 4-chloro-1-naftol, 15 ml methyalcohol, and 60 ml 10 mM Tris-HCl pH 6.8) by drop-wise addition of a 25 μl aliquot of hydrogen peroxide (H_2_O_2_, up to 100 μl) until membrane saturation.

### Microscopy

Pollen tube bundles from excised pistils were mounted directly on a glass slide and visualized by bright field microscopy with Hoffman modulation contrast (Nikon, Japan). For aniline blue staining (callose stain), pistils were collected 18 HAP and fixed in 9:1 ethanol:acetic acid (v/v) for 24 h. Samples were washed in an ethanol series and then alkaline-treated in 1.0 M NaOH for ~16 h. Samples were stained in aniline blue stain solution (0.1 % (w/v) aniline blue, 108 mM K_3_PO_4_ (pH 11)) for 12 h. To investigate the competence of pollen tubes to target the ovule by blue dot GUS-staining assay, pistils pollinated with +/*tctp-1* SAIL_28_C03 or Lat52-GUS-marked wild-type control pollen were collected at 18 HAP and dissected along the septum to remove carpel walls. Exposed fertilized ovules were stained for GUS activity with a solution containing 50 mM sodium phosphate buffer, pH 7, 0.2 % Triton X-100, 10 mM potassium ferrocyanide, 10 mM potassium ferricyanide, and 1 mM X-Gluc (5-bromo-4-chloro-3-indolyl-D-glucoronic acid). Samples were vacuum-infiltrated for 10 min and stained overnight at 37 °C.

For the pollen tube viability test using Alexander staining, pollen tubes were placed on a microscopic slide with a few drops of Alexander stain solution (10 ml 95 % ethanol, 1 ml Malachite green (1 % in 95 % ethanol), 5 ml Fuchsin acid (1 % in water), 0.5 ml Orange G (1 % in water), 5 g phenol, 5 g chloral hydrate, 2 ml glacial acetic acid, 25 ml glycerol, and distilled water to 50 ml). Stained samples were visualized by bright field microscopy with Hoffman modulation contrast (Nikon, Japan). Callose stain distribution was analyzed with NIS-Element software following fixed UV exposure under cyan filter (460 − 500 nm bandwidth, Nikon, Japan). For propidium iodide staining, tissues were incubated in 10 μg/ml propidium iodide for 20 s, rinsed in deionized water, and visualized under a RFP filter (560–615 nm, Nikon, Japan).

For protein subcellular localization, samples were visualized with a Zeiss LSM 5 DUO laser scanning confocal microscope (CLSM) equipped with an argon laser and a Zeiss C-Apochromat × 40 9/1.2 water-corrected objective. For co-localization, dual fluorescence channels and differential interference contrast (DIC) were used simultaneously for live cell imaging. A Nikon Eclipse Ti confocal microscope with a CSU-X1 spinning disk module and Andor iXon3 EMCCD camera was used to verify vesicle dynamics as well as subcellular localization in pollen tubes. Images were analyzed and assembled with ImageJ (http://imagej.net), Adobe Photoshop CS6 (http://www.adobe.com/) and Ink-scape (https://inkscape.org/en/) software.

### Availability of supporting raw data

The mass spectrometry proteomics data sets supporting the results of this article are available at the ProteomeXchange Consortium via the PRIDE partner repository with the dataset identifier PXD002215 (http://www.ebi.ac.uk/pride/archive/projects/PXD002215).

## Additional files

Additional file 1: Figure S1. Semi-in vivo pollen tube secretome (SIV-PS) approach for identification and quantification of pollen tube-secreted proteins. a An improvised SIV-PS technique setup from *in planta* (steps 1–3) to in vitro incubation of the pollen tubes (step 4–5). The *inset* shows emerging pollen tubes from excised pistils. Scale bars = 2 mm. b Schematic representation of the SIV-PS workflow. c Micrographs of SIV pollen tubes showing normal pollen tube growth in bright field with streaming organelles (*top panel*), sperm cell formation (*second panel*), callose deposition and callose plugs (*asterisk*, *third panel*), and pollen tube viability assessed by Alexander stain (*bottom panel*). *sn* sperm cell nucleus, *VN* vegetative cell nucleus. Scale bar = 40 μM. d A tobacco pollinated pistil showing the site of stylar excision and the presumed peptide signaling flow from male and female gametophytes. (TIF 714 kb) Additional file 2: Figure S2. Assessment of pollen tube integrity, secretome purity using an alcohol-dehydrogenase (ADH) assay, and secretion activities in pollen tubes. a *Top panels*: pollen tube penetration (*left*) and exit (*right*) through the transmitting tract of the style visualized with aniline blue. *Bottom panels*: excised stylar ends visualized by DAPI stain marking cell nuclei (*asterisk*, *left*) and by toluidine blue (*right*) outlining cell wall boundaries. Excised ends of pistils are marked with the *dashed line*; *red* and *white arrowheads* mark the upper border of pistil broken cells following excision; and *yellow arrowheads* mark the upper and lower borders of intact cells. Scale bars = 5 μM. b Frequency of viable and non-viable semi*-*in vivo pollen tubes as well as integrity following 48 h growth through stylar tissues and in vitro. Error bars represent ± standard error; *asterisks* indicate statistical significance by Student’s *t*-test with *p* values indicated. c Purity assessment using an ADH assay of the secretome samples. ADH activities were calculated as nmole/min/ml. *SIV-PS1–3* SIV-pollen tube secretome samples 1–3. d–f Live-cell imaging of endocytic tracer FM4-64 uptake and recycling in tobacco in vitro germinated pollen tubes co-localized with ER tracer in the absence (d) or presence (e, f) of brefeldin A (*BFA*), an inhibitor of protein secretion. Addition of BFA resulted in aggregation of endocytic vesicles and formation of BFA compartments (*arrows*), evidence of a defective conventional protein secretion pathway. BFA-treated pollen tubes showed specific defects only in FM4-64 labeled vesicles and not in the ER network. Scale bars = 5 μM. (TIF 1554 kb) Additional file 3: Table S1. List of protein accessions identified from semi*-*in vivo pollen tube secretome and semi-in vivo total proteomes by gel-free LC/MS/MS. (XLSX 53714 kb) Additional file 4: Figure S3. Evaluation of true pollen tube-secreted proteins using quantitative LC-MS/MS. Depicted are three examples of proteins categorized as pollen tube-secreted proteins or predominantly secreted by the pollen tube following peptide mapping and quantitative peptide area evaluation to compute protein abundances relative to control. With subtractive approaches, these proteins would normally be eliminated from analysis as they were also identified in unpollinated pistil controls (most likely due to peptide homology with pistil proteins); however, using absolute quantitative analysis, their true source of secretion could clearly be demonstrated. (TIF 415 kb) Additional file 5: Table S2. Tobacco semi-in vivo pollen tube secreted proteins of ≤ 20 kDa. (XLSX 306 kb) Additional file 6: Table S3. Identified protein families and domains of semi-in vivo pollen tube-secreted proteins. (XLSX 515 kb) Additional file 7: Table S4. List of protein accessions derived from protein supergroup (see materials and methods) pairwise comparison listing unique and common protein accessions between SIV-PS and in vitro pollen tube secretomes and proteomes. The data file also includes individual replicates of in vitro pollen tube-secreted proteins and identified total proteomes. (XLSX 30434 kb) Additional file 8: Table S5. Pollen tube secretome protein accessions mapped to the Arabidopsis semi-in vivo transcriptome. (XLSX 178 kb) Additional file 9: Figure S4. Bioinformatic workflow and detection of N-glycosylation of secreted proteins by concavalin A staining. a In silico classification of secreted proteins into conventionally and unconventionally secreted proteins based on the presence or absence of an N-terminal signal peptide (SP) using SignalP v4.1 prediction algorithms. Each protein class was further analyzed for its potential secretion using the secretomeP database and putative post-translational modifications as indicated. b Prediction of plasma membrane GPI-anchoring using FragAnchor. The reliability of the implemented prediction algorithms was tested by independently analyzing Uniprot-derived accessions (*upper column*) and in-house *Nicotiana tabacum* protein sequences (*lower column*) with limited sequence annotation. c Subcellular localization prediction using the LocTree prediction database. Independent prediction with the targetP database (Additional file 10: Figure S5) emphasized secretion of SP-containing proteins and largely ambiguous localization of unconventionally secreted pollen tube proteins. d One-dimensional SDS-PAGE profiling of pollen tube-secreted proteins. *Right*: banding patterns show the distinct profile of SIV secreted proteins (SIV-PS1, SIV-PS3) relative to unpollinated pistil control. e Concavalin A glycosylated protein detection of pollen tube-secreted proteins. A subfraction of SIV-PS is postranslationally glycosylated (*red rectangle*). (TIF 896 kb) Additional file 10: Figure S5. Prediction of transmembrane helices (TMHs) in pollen tube-secreted proteins and LocTree-associated GO terms. a Analysis of TMHs of pollen tube-secreted proteins using TMHMM algorithms. *Right*: validation of TMHMM algorithms using sequences from known *Arabidopsis* pollen tube plasma membrane proteins (ACA9, ANX1, ANX2, KRP2, KRP4) and non-TMH-containing pollen tube palmitoylated plasma membrane proteins LIP1 and LIP2 as controls. The absence of TMHs in pollen tube-secreted proteins also supports their potential secretion to the apoplast and extracellular matrix. b Independent prediction of pollen tube-secreted protein subcellular localization using the TargetP database. c Representative GO terms associated with the corresponding LocTree-predicted localization (Additional file 9: Figure S4) derived from PSI-Blast of pollen tube-secreted proteins. (TIF 780 kb) Additional file 11: Figure S6. Pollen tube secretome GO-slim term enrichment and expression profiling of secretory pathway genes. a Enriched GO-slim term comparison. The color scale indicates comparative enrichment and overlaid bar charts show GO term enrichment within protein subgroups; numbers represent associated accessions (*p* < 0.05). b Unique GO-slim terms of the unconventionally secreted pollen tube protein subset. A full list of GO terms is provided in Additional file 8: Table S5. c Plant secretory pathways with annotated genes derived from genetic studies [79–81]. d Microarray profiling of secretory pathway genes during tobacco pollen tube growth. e Semi-RT-PCR validation of selected secretory pathway genes in tobacco semi*-*in vivo pollen tubes and unfertilized ovules. A list of primers is provided in Additional file 20: Table S8. (TIF 610 kb) Additional file 12: Figure S7. Assessment of pollen tube secreted RNases and defensin subgroup protein homology. a PD1 RNase family protein alignment with tobacco-derived NtRNase1 and RNase NE proteins. b RT-PCR analysis of pollen tube-secreted RNases showing specific expression in female reproductive tissues alone in unpollinated as well as pollinated samples (14 hours post-pollination). *PT24* 24 h cultivated in vitro pollen tubes. c Alignment of *Torenia fournieri* CRP1–3 with NtPsCRP1. *Pink highlighting* indicates the predicted signal peptide of NtPsCRP1 and the *green highlighting* shows the six conserved cysteine residues of the defensin subfamily. Amino acids conserved in at least two sequences are shaded. d Neighbor-joining phylogenetic tree using percentage identity and node values showing closer association of NtPsCRP1 with TfCRP1 and TfCRP3. A list of primers used is provided in Additional file 20: Table S8. (TIF 546 kb) Additional file 13: Figure S8. Pairwise alignment of *Arabidopsis* and tobacco TCTP proteins and expression profile of LAT52 and PRK2/4 receptor kinases. a Amino acid conservation between the *Arabidopsis* and *N. tabacum* TCTP protein. *Blue highlighting* indicates mismatches. b A hydropathy plot of NtTCTP hydrophobicity prediction based on the Kyte Doolitle method showing GRAVY score (*red line*) and overall hydrophobic nature of NtTCTP. c In vitro pollen tube growth assay of +/*Attctp-1* tetrad pollen showing normal pollen tube germination. d Aniline blue staining of self-fertilized +/*Attctp-1* pistils 18 h after pollination, independently verifying normal pollen tube growth *in planta*. e Pairwise alignment of pollen tube-secreted *L. barbarum* LAT52-like and *S. lycopersicum* LAT52 proteins. Alignment showing the predicted 23 amino acid N-terminal signal peptide (*blue highlighting*), the conserved eight cysteine residues (*boxed*), and N-glycosylation sites (*grey highlighting*). f Expression profiles of pollen receptor kinase 2 (PRK2) and pollen receptor kinase 4 (PRK4) derived from Agilent 44 K Tobacco Genome Array [19] and Arabidopsis Affymetrix ATH1 microarray [53] data. g Semi-RT-PCR verification of PRK2 and PRK4 expression in tobacco mature pollen (*MP*), 4 h (*PT4*) and 24 h (*PT24*) in vitro pollen tubes, leaves (*LF*), roots (*RT*), SIV pollen tubes, and unfertilized ovules. h Topology of the receptor modules, PRK2 and PRK4, and the ligand module LAT52. We speculate that detection of both modules after 24 h of pollen tube growth implies a later function of the complex leading to successful fertilization. (TIF 953 kb) Additional file 14: Figure S9. Secreted NtPsCRP2 displays “stop-and-go” movements in root epidermal cells. a A 2’30” span confocal time-series and b a close-up 0’55” of the junction of two root epidermal cells showing NtPsCRP2-GFP localized activities at the root elongation zone. Three classes of activities could be deduced: “a”, plasma membrane tethered or dormant (static) vesicles; “b”, tethered at first followed by active movements; and “c”, mobile at first followed by tethering. Scale bars = 5 μM. (TIF 1515 kb) Additional file 15: Movie 1. The “stop-and-go” cycling movements of NtPsCRP2-GFP in *Arabidopsis* roots. (AVI 17600 kb) Additional file 16: Movie 2. A close-up of NtPsCRP2-GFP “stop-and-go” movements at the junction of two epidermal cells at the root elongation zone. (AVI 3679 kb) Additional file 17: Table S6. GO terms enriched in the semi-in vivo pollen tube secretome. (XLSX 472 kb) Additional file 18: Table S7. Overlap between the tobacco semi-in vivo pollen tube secretome and the proteome of olive pollen tube-secreted nanovesicle exosomes. (XLSX 17 kb) Additional file 19: Figure S10. Microarray expression profiling of AtLLG3 pre- and post-pollination. a High abundance of AtLLG3 in mature pollen relative to whole flower expression. b A near twofold increase of AtLLG3 expression in pollinated pistils 8 h after pollination relative to 3.5 h post-pollination. Expression data were derived from the Affymetrix Arabidopsis ATH1 Genome Array at Genevestigator [53]. c Predicted NtLLG3 25 amino acid N-terminal signal peptide motif (*red*) as well as protease cleavage site (*arrow*). At the C-terminus is the predicted GPI-anchor site (*blue*) and cleavage site (serine, *red underlined S*) for anchor release. (TIF 402 kb) Additional file 20. Supplementary text. Expression profiling of core genes of the classic secretory pathways in semi-in vivo pollen tubes and unfertilized ovules. (DOC 23 kb) Additional file 21: Table S8. List of primers used in this study. (XLSX 12 kb)

## Abbreviations

ADH: alcohol dehydrogenase
BFA: brefeldin A
CRP: cysteine-rich protein
DEFL: defensin-like
ER: endoplasmic reticulumn
FDR: false discovery rate
GAP: GPI-anchored protein
GFP: green fluorescent protein
GO: Gene Ontology
GPI: glycophosphatidylinositol
HAP: hours after pollination
LC: liquid chromatography
LLG3: Lorelei-like GPI-anchored protein 3
LTP: lipid transfer protein
MS: mass spectrometry
MS/MS: tandem mass spectrometry
PEG: polyethylene glycol
ppm: parts per million
PT24-PP: 24 h in vitro pollen tube proteome
PT24-PS: 24 h in vitro pollen tube secretome
RFP: red fluorescent protein
SIV: semi in vivo
SIV-PS: semi-in vivo pollen tube secretome
SP: signal peptide
TCTP: Translationally controlled tumor protein
TGN: trans-Golgi network
TMH: transmembrane helix
YIP4a/b: YPT/RAB GTPase interacting protein 4a/b
