# Supplementary figures and images for: Quantitative proteomics of the tobacco pollen tube secretome identifies novel pollen tube guidance proteins important for fertilization
Source: Genome Biol. 2016 May 3;17:81. doi: 10.1186/s13059-016-0928-x (PMC4853860; doi:10.1186/s13059-016-0928-x)

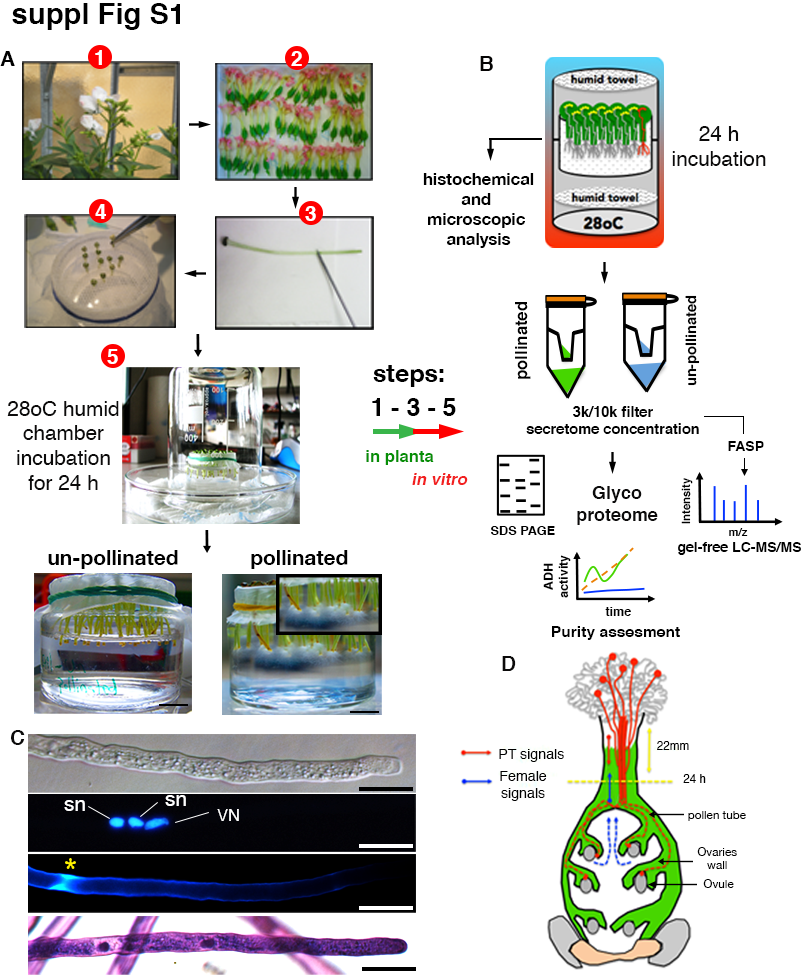

Supplement: Additional file 1: Figure S1. — Semi-in vivo pollen tube secretome (SIV-PS) approach for identification and quantification of pollen tube-secreted proteins. a An improvised SIV-PS technique setup from in planta (steps 1–3) to in vitro incubation of the pollen tubes (step 4–5). The inset shows emerging pollen tubes from excised pistils. Scale bars = 2 mm. b Schematic representation of the SIV-PS workflow. c Micrographs of SIV pollen tubes showing normal pollen tube growth in bright field with streaming organelles (top panel), sperm cell formation (second panel), callose deposition and callose plugs (asterisk, third panel), and pollen tube viability assessed by Alexander stain (bottom panel). sn sperm cell nucleus, VN vegetative cell nucleus. Scale bar = 40 μM. d A tobacco pollinated pistil showing the site of stylar excision and the presumed peptide signaling flow from male and female gametophytes. (TIF 714 kb) [file 13059_2016_928_MOESM1_ESM.tif]

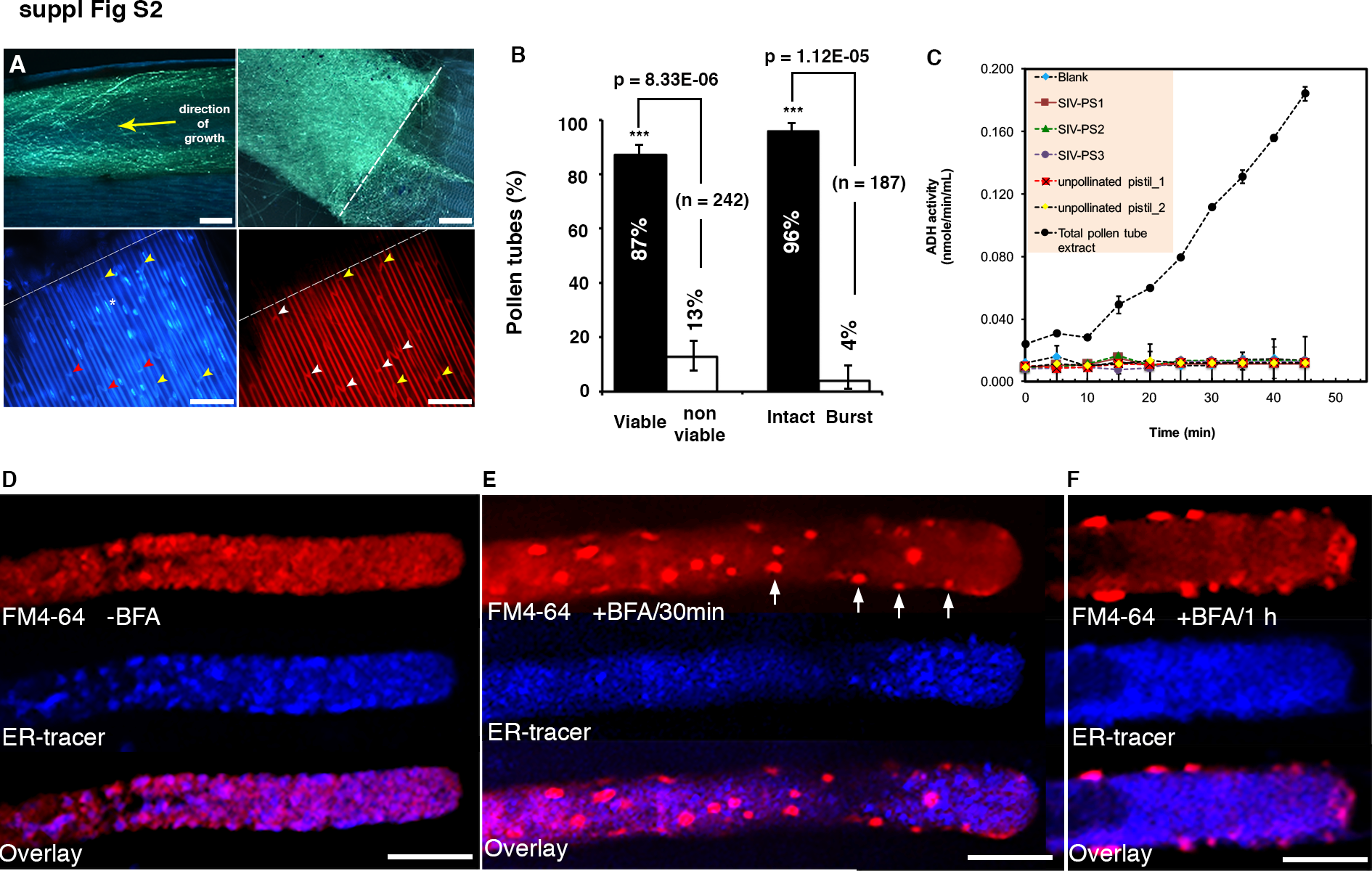

Supplement: Additional file 2: Figure S2. — Assessment of pollen tube integrity, secretome purity using an alcohol-dehydrogenase (ADH) assay, and secretion activities in pollen tubes. a Top panels: pollen tube penetration (left) and exit (right) through the transmitting tract of the style visualized with aniline blue. Bottom panels: excised stylar ends visualized by DAPI stain marking cell nuclei (asterisk, left) and by toluidine blue (right) outlining cell wall boundaries. Excised ends of pistils are marked with the dashed line; red and white arrowheads mark the upper border of pistil broken cells following excision; and yellow arrowheads mark the upper and lower borders of intact cells. Scale bars = 5 μM. b Frequency of viable and non-viable semi-in vivo pollen tubes as well as integrity following 48 h growth through stylar tissues and in vitro. Error bars represent ± standard error; asterisks indicate statistical significance by Student’s t-test with p values indicated. c Purity assessment using an ADH assay of the secretome samples. ADH activities were calculated as nmole/min/ml. SIV-PS1–3 SIV-pollen tube secretome samples 1–3. d–f Live-cell imaging of endocytic tracer FM4-64 uptake and recycling in tobacco in vitro germinated pollen tubes co-localized with ER tracer in the absence (d) or presence (e, f) of brefeldin A (BFA), an inhibitor of protein secretion. Addition of BFA resulted in aggregation of endocytic vesicles and formation of BFA compartments (arrows), evidence of a defective conventional protein secretion pathway. BFA-treated pollen tubes showed specific defects only in FM4-64 labeled vesicles and not in the ER network. Scale bars = 5 μM. (TIF 1554 kb) [file 13059_2016_928_MOESM2_ESM.tif]

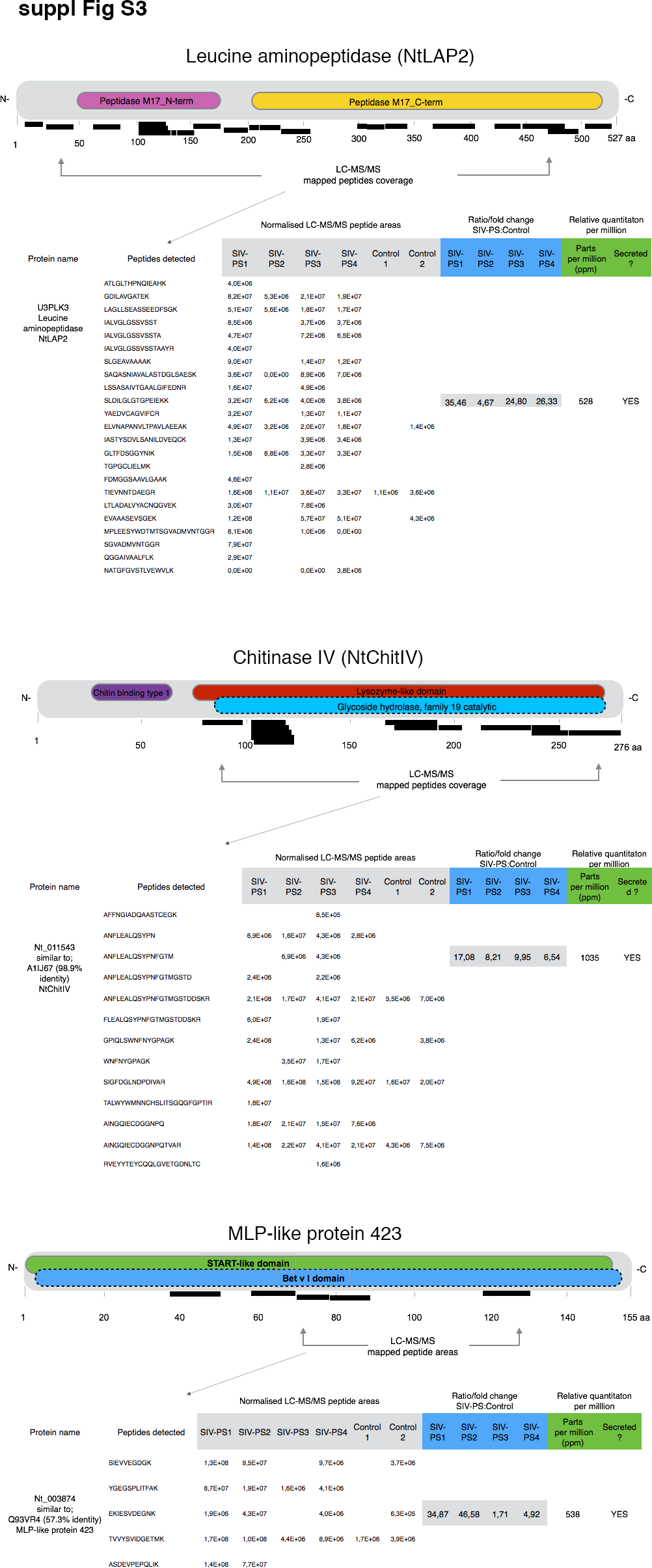

Supplement: Additional file 4: Figure S3. — Evaluation of true pollen tube-secreted proteins using quantitative LC-MS/MS. Depicted are three examples of proteins categorized as pollen tube-secreted proteins or predominantly secreted by the pollen tube following peptide mapping and quantitative peptide area evaluation to compute protein abundances relative to control. With subtractive approaches, these proteins would normally be eliminated from analysis as they were also identified in unpollinated pistil controls (most likely due to peptide homology with pistil proteins); however, using absolute quantitative analysis, their true source of secretion could clearly be demonstrated. (TIF 415 kb) [file 13059_2016_928_MOESM4_ESM.tif]

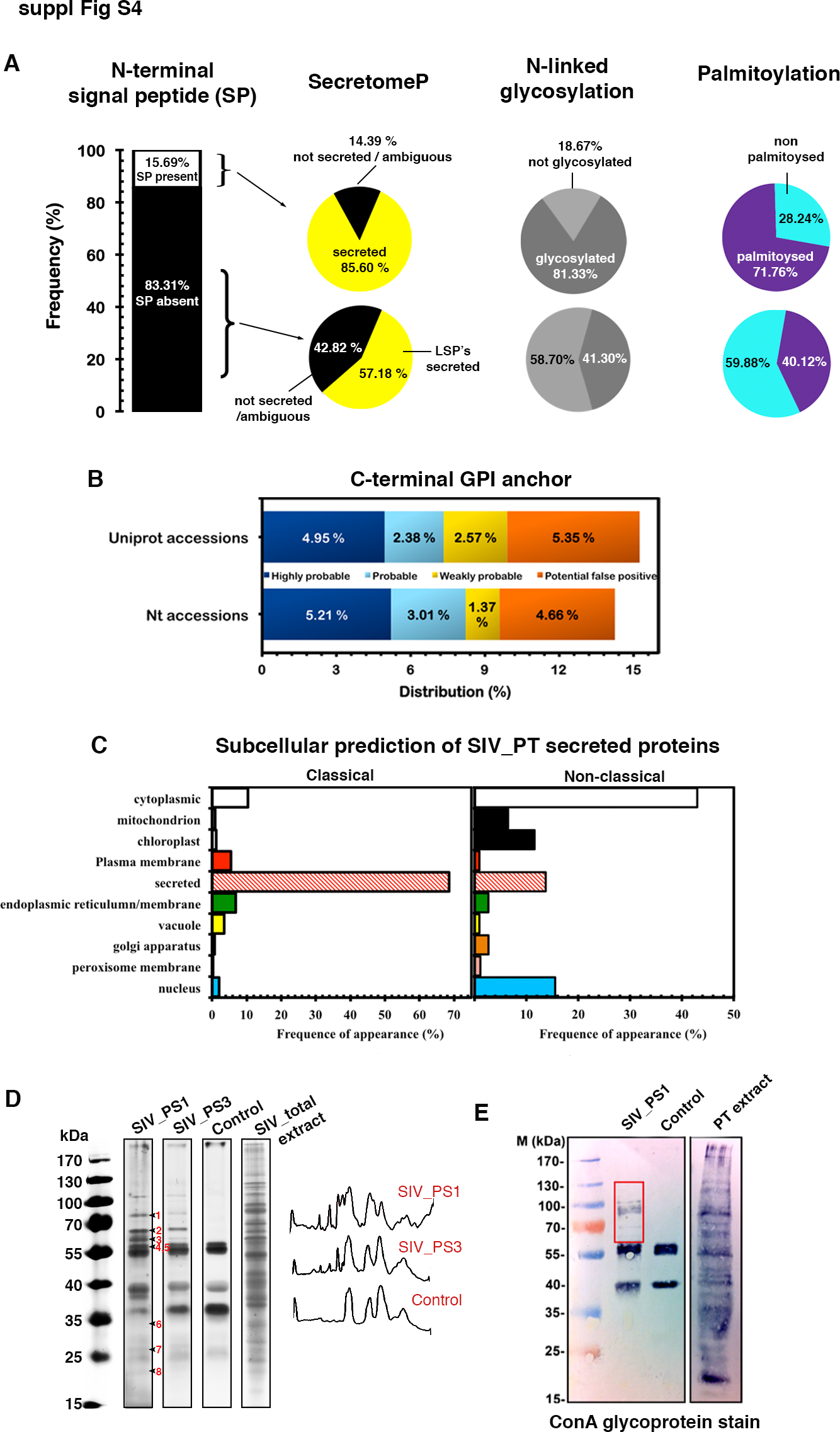

Supplement: Additional file 9: Figure S4. — Bioinformatic workflow and detection of N-glycosylation of secreted proteins by concavalin A staining. a In silico classification of secreted proteins into conventionally and unconventionally secreted proteins based on the presence or absence of an N-terminal signal peptide (SP) using SignalP v4.1 prediction algorithms. Each protein class was further analyzed for its potential secretion using the secretomeP database and putative post-translational modifications as indicated. b Prediction of plasma membrane GPI-anchoring using FragAnchor. The reliability of the implemented prediction algorithms was tested by independently analyzing Uniprot-derived accessions (upper column) and in-house Nicotiana tabacum protein sequences (lower column) with limited sequence annotation. c Subcellular localization prediction using the LocTree prediction database. Independent prediction with the targetP database (Additional file 10: Figure S5) emphasized secretion of SP-containing proteins and largely ambiguous localization of unconventionally secreted pollen tube proteins. d One-dimensional SDS-PAGE profiling of pollen tube-secreted proteins. Right: banding patterns show the distinct profile of SIV secreted proteins (SIV-PS1, SIV-PS3) relative to unpollinated pistil control. e Concavalin A glycosylated protein detection of pollen tube-secreted proteins. A subfraction of SIV-PS is postranslationally glycosylated (red rectangle). (TIF 896 kb) [file 13059_2016_928_MOESM9_ESM.tif]

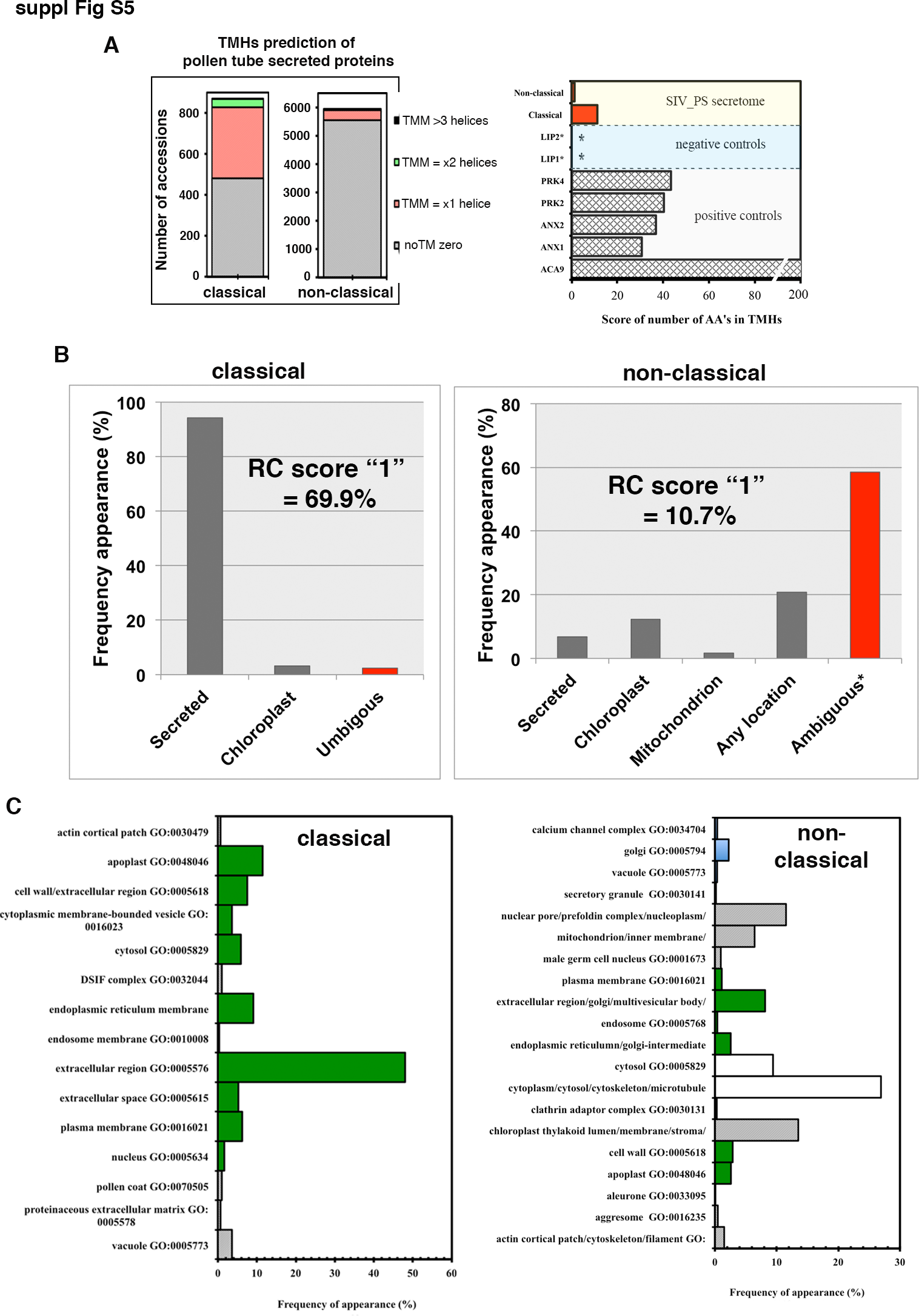

Supplement: Additional file 10: Figure S5. — Prediction of transmembrane helices (TMHs) in pollen tube-secreted proteins and LocTree-associated GO terms. a Analysis of TMHs of pollen tube-secreted proteins using TMHMM algorithms. Right: validation of TMHMM algorithms using sequences from known Arabidopsis pollen tube plasma membrane proteins (ACA9, ANX1, ANX2, KRP2, KRP4) and non-TMH-containing pollen tube palmitoylated plasma membrane proteins LIP1 and LIP2 as controls. The absence of TMHs in pollen tube-secreted proteins also supports their potential secretion to the apoplast and extracellular matrix. b Independent prediction of pollen tube-secreted protein subcellular localization using the TargetP database. c Representative GO terms associated with the corresponding LocTree-predicted localization (Additional file 9: Figure S4) derived from PSI-Blast of pollen tube-secreted proteins. (TIF 780 kb) [file 13059_2016_928_MOESM10_ESM.tif]

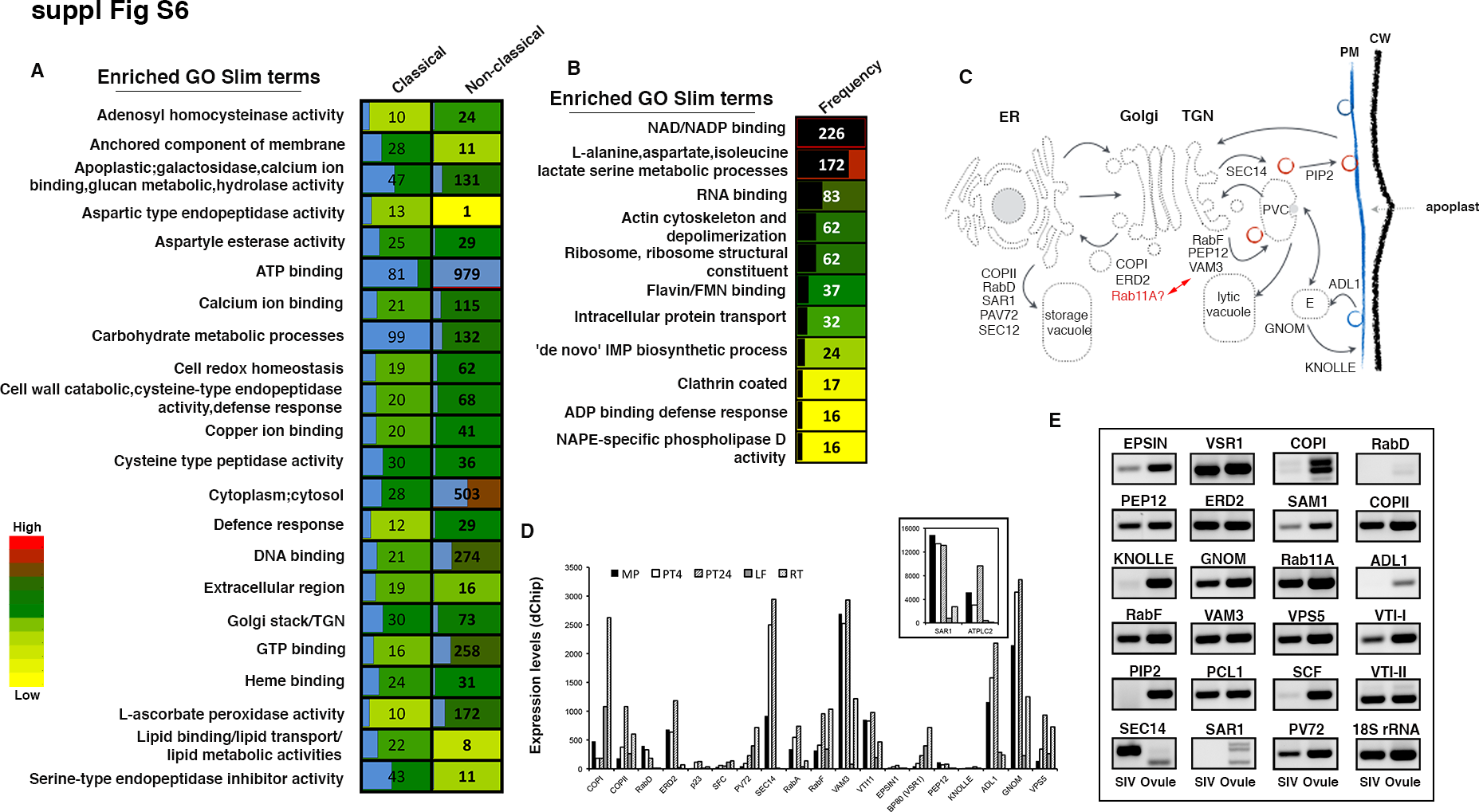

Supplement: Additional file 11: Figure S6. — Pollen tube secretome GO-slim term enrichment and expression profiling of secretory pathway genes. a Enriched GO-slim term comparison. The color scale indicates comparative enrichment and overlaid bar charts show GO term enrichment within protein subgroups; numbers represent associated accessions (p < 0.05). b Unique GO-slim terms of the unconventionally secreted pollen tube protein subset. A full list of GO terms is provided in Additional file 8: Table S5. c Plant secretory pathways with annotated genes derived from genetic studies [79–81]. d Microarray profiling of secretory pathway genes during tobacco pollen tube growth. e Semi-RT-PCR validation of selected secretory pathway genes in tobacco semi-in vivo pollen tubes and unfertilized ovules. A list of primers is provided in Additional file 20: Table S8. (TIF 610 kb) [file 13059_2016_928_MOESM11_ESM.tif]

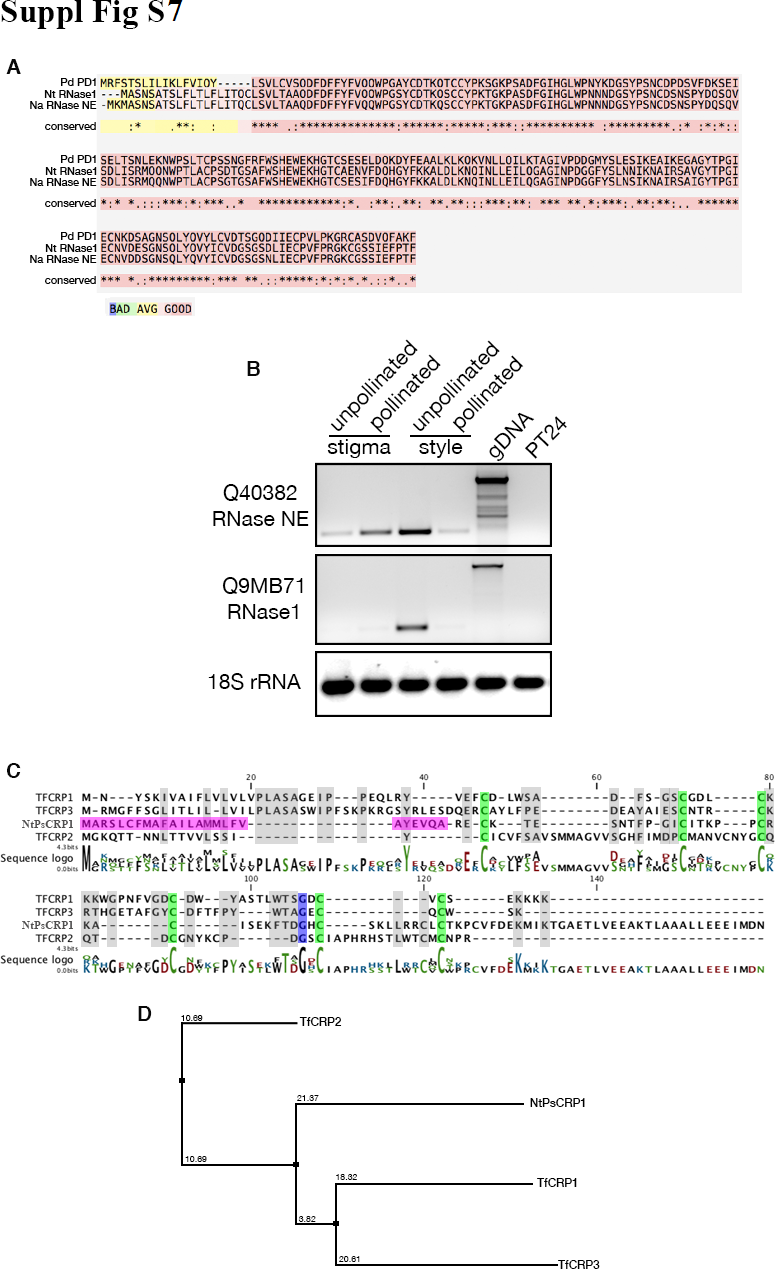

Supplement: Additional file 12: Figure S7. — Assessment of pollen tube secreted RNases and defensin subgroup protein homology. a PD1 RNase family protein alignment with tobacco-derived NtRNase1 and RNase NE proteins. b RT-PCR analysis of pollen tube-secreted RNases showing specific expression in female reproductive tissues alone in unpollinated as well as pollinated samples (14 hours post-pollination). PT24 24 h cultivated in vitro pollen tubes. c Alignment of Torenia fournieri CRP1–3 with NtPsCRP1. Pink highlighting indicates the predicted signal peptide of NtPsCRP1 and the green highlighting shows the six conserved cysteine residues of the defensin subfamily. Amino acids conserved in at least two sequences are shaded. d Neighbor-joining phylogenetic tree using percentage identity and node values showing closer association of NtPsCRP1 with TfCRP1 and TfCRP3. A list of primers used is provided in Additional file 20: Table S8. (TIF 546 kb) [file 13059_2016_928_MOESM12_ESM.tif]

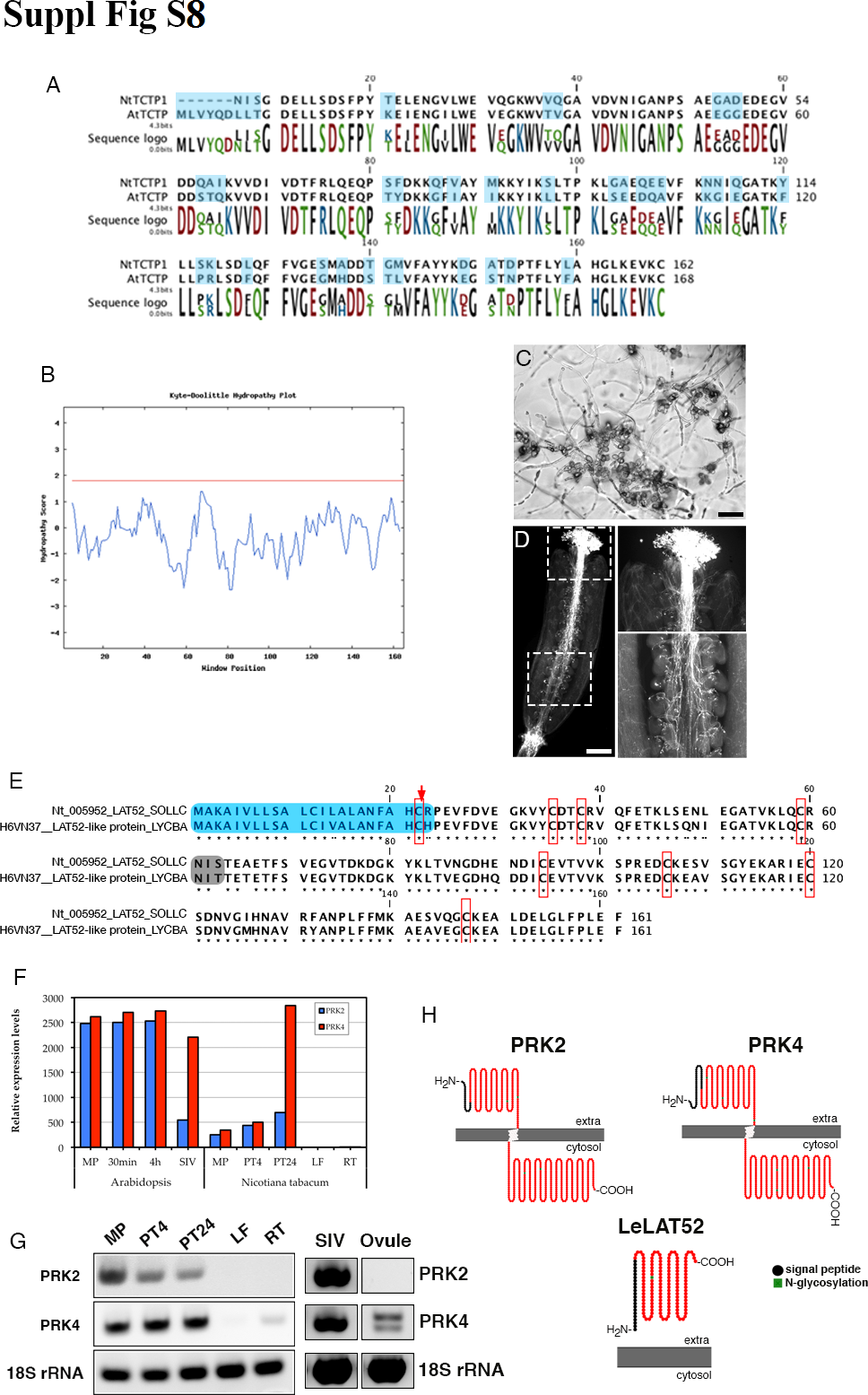

Supplement: Additional file 13: Figure S8. — Pairwise alignment of Arabidopsis and tobacco TCTP proteins and expression profile of LAT52 and PRK2/4 receptor kinases. a Amino acid conservation between the Arabidopsis and N. tabacum TCTP protein. Blue highlighting indicates mismatches. b A hydropathy plot of NtTCTP hydrophobicity prediction based on the Kyte Doolitle method showing GRAVY score (red line) and overall hydrophobic nature of NtTCTP. c In vitro pollen tube growth assay of +/Attctp-1 tetrad pollen showing normal pollen tube germination. d Aniline blue staining of self-fertilized +/Attctp-1 pistils 18 h after pollination, independently verifying normal pollen tube growth in planta. e Pairwise alignment of pollen tube-secreted L. barbarum LAT52-like and S. lycopersicum LAT52 proteins. Alignment showing the predicted 23 amino acid N-terminal signal peptide (blue highlighting), the conserved eight cysteine residues (boxed), and N-glycosylation sites (grey highlighting). f Expression profiles of pollen receptor kinase 2 (PRK2) and pollen receptor kinase 4 (PRK4) derived from Agilent 44 K Tobacco Genome Array [19] and Arabidopsis Affymetrix ATH1 microarray [53] data. g Semi-RT-PCR verification of PRK2 and PRK4 expression in tobacco mature pollen (MP), 4 h (PT4) and 24 h (PT24) in vitro pollen tubes, leaves (LF), roots (RT), SIV pollen tubes, and unfertilized ovules. h Topology of the receptor modules, PRK2 and PRK4, and the ligand module LAT52. We speculate that detection of both modules after 24 h of pollen tube growth implies a later function of the complex leading to successful fertilization. (TIF 953 kb) [file 13059_2016_928_MOESM13_ESM.tif]

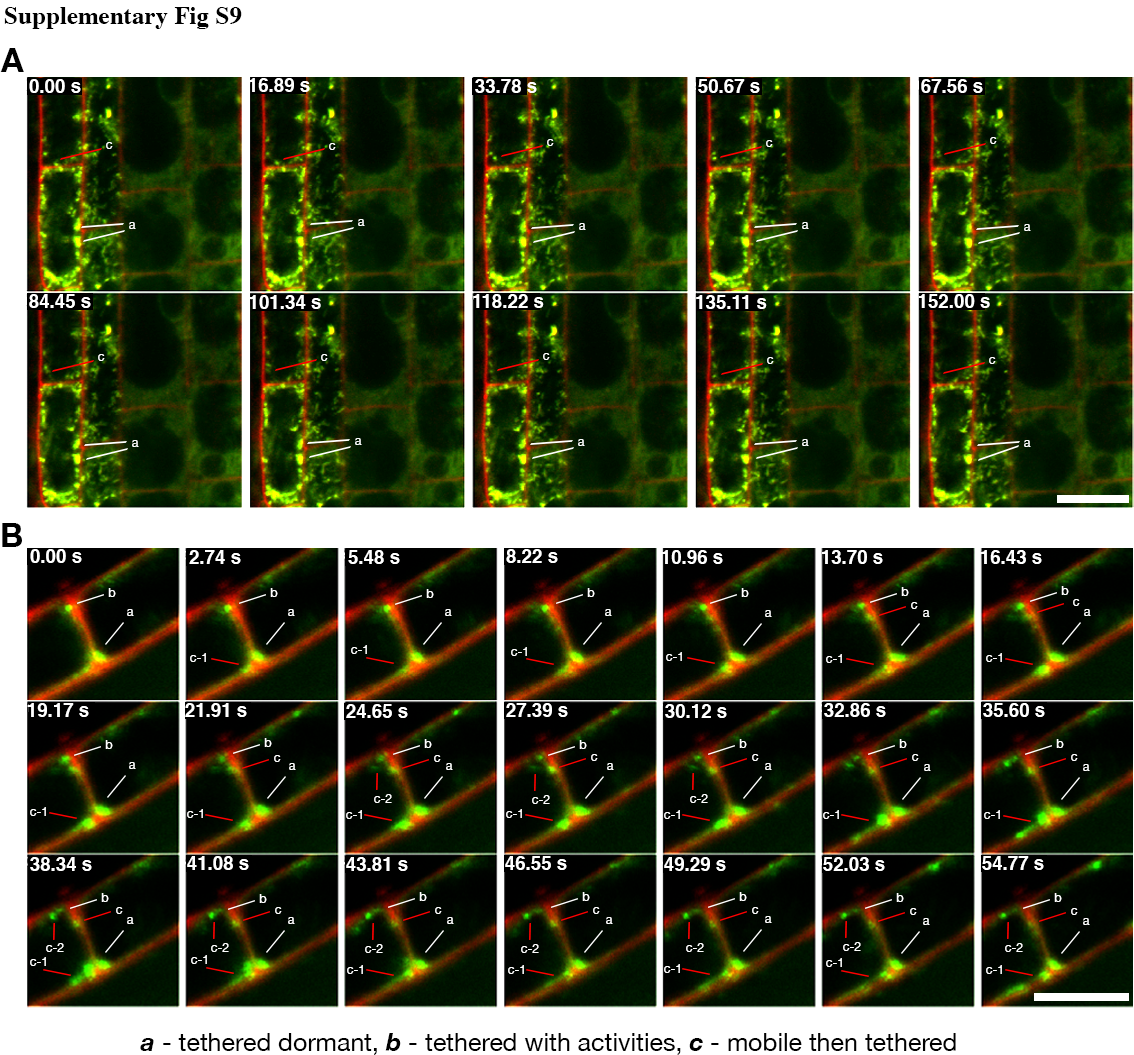

Supplement: Additional file 14: Figure S9. — Secreted NtPsCRP2 displays “stop-and-go” movements in root epidermal cells. a A 2’30” span confocal time-series and b a close-up 0’55” of the junction of two root epidermal cells showing NtPsCRP2-GFP localized activities at the root elongation zone. Three classes of activities could be deduced: “a”, plasma membrane tethered or dormant (static) vesicles; “b”, tethered at first followed by active movements; and “c”, mobile at first followed by tethering. Scale bars = 5 μM. (TIF 1515 kb) [file 13059_2016_928_MOESM14_ESM.tif]

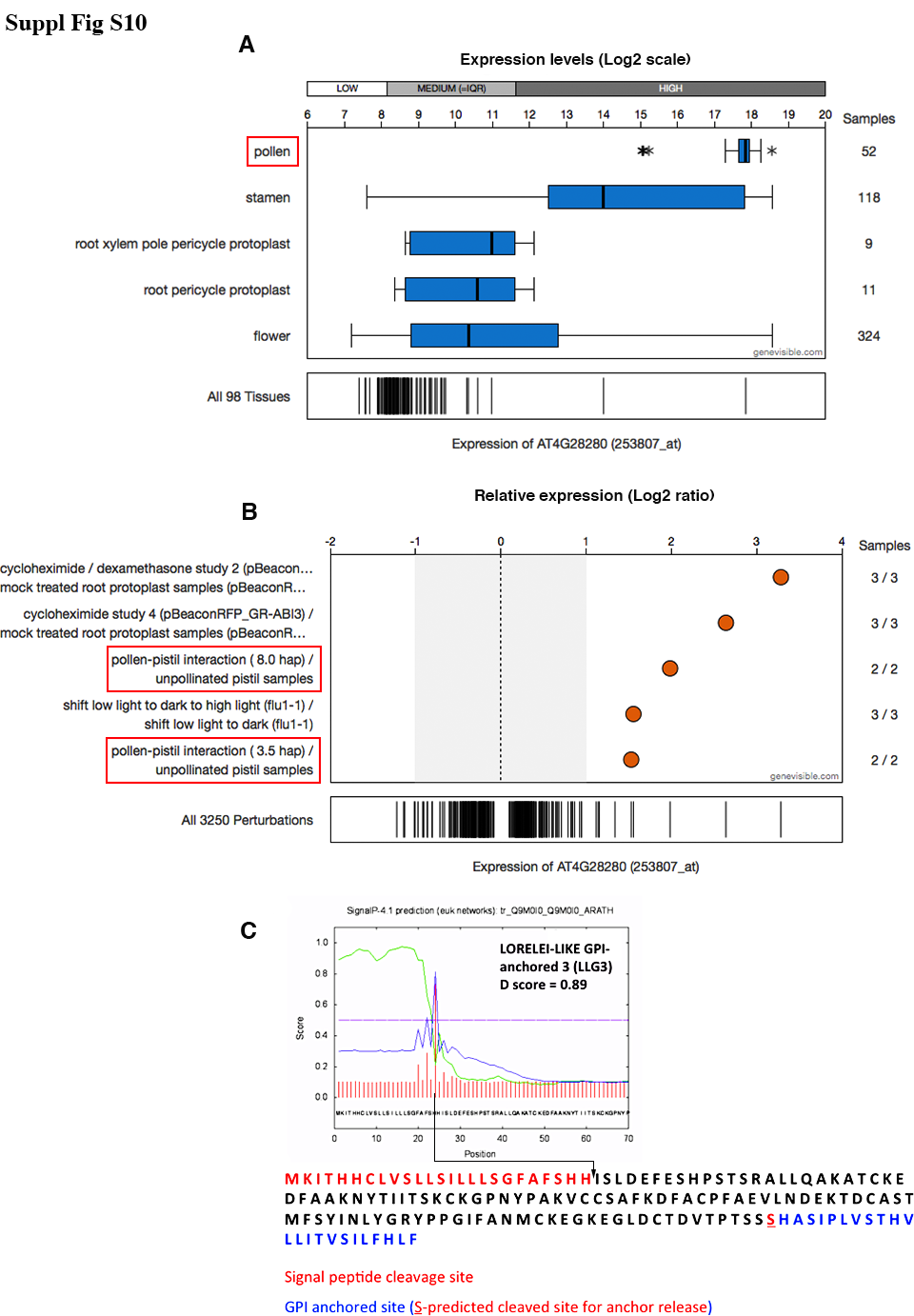

Supplement: Additional file 19: Figure S10. — Microarray expression profiling of AtLLG3 pre- and post-pollination. a High abundance of AtLLG3 in mature pollen relative to whole flower expression. b A near twofold increase of AtLLG3 expression in pollinated pistils 8 h after pollination relative to 3.5 h post-pollination. Expression data were derived from the Affymetrix Arabidopsis ATH1 Genome Array at Genevestigator [53]. c Predicted NtLLG3 25 amino acid N-terminal signal peptide motif (red) as well as protease cleavage site (arrow). At the C-terminus is the predicted GPI-anchor site (blue) and cleavage site (serine, red underlined S) for anchor release. (TIF 402 kb) [file 13059_2016_928_MOESM19_ESM.tif]
