# Supplementary material for: Quantitative proteomics of the tobacco pollen tube secretome identifies novel pollen tube guidance proteins important for fertilization
Source: Genome Biol. 2016 May 3;17:81. doi: 10.1186/s13059-016-0928-x (PMC4853860; doi:10.1186/s13059-016-0928-x)
Supplement: Additional file 20. — Supplementary text. Expression profiling of core genes of the classic secretory pathways in semi-in vivo pollen tubes and unfertilized ovules. (DOC 23 kb) [file 13059_2016_928_MOESM20_ESM.doc]

**Supplementary text**

**Expression profiling of core genes of the classical secretory pathways in in *semi in vivo* pollen tubes and unfertilized ovules**

We have analysed transcripts abundance of genes regulating conventional protein secretion with tobacco Agilent 44K Genome Array expression data [19,20] and found genes involved in ER-Golgi transport (COPI and COPII), retrograde Golgi-vesicles (VSR1 and PEP12) as well as pre-vacuolar formation and vesicles membrane fusion (SEC14) to be expressed in mature pollen (MP) and in pollen tubes cultivated *in vitro* up to 24 h (Figure S6C-E). Intriguingly, among the genes investigated, 10/21 were significantly more abundant in pollen and pollen tubes than in leaves and root tissues (Figure S6D). Moreover, SAR1 and PLC2 transcripts were 4-6 fold higher in the male gametophyte than in sporophyte as well as in comparison to other genes of the classical secretory pathway. However, we could not verify SAR1 expression by semi RT-PCR in *semi in vivo* pollen tubes and PLC2 lacked specific primers for PCR test (Figure S6E). Semi RT-PCR analysis of additional known components of the classical secretory pathway showed consistent expression profile to that of microarray and variable transcripts abundance in SIV pollen tubes as well as unfertilized ovules (Figure S6E). Activities of these core genes are likely to regulate pollen tube secretion of proteins detected in this study.
